# High levels of glucose alter *Physcomitrella patens* metabolism and trigger a differential proteomic response

**DOI:** 10.1371/journal.pone.0242919

**Published:** 2020-12-04

**Authors:** Alejandra Chamorro-Flores, Axel Tiessen-Favier, Josefat Gregorio-Jorge, Miguel Angel Villalobos-López, Ángel Arturo Guevara-García, Melina López-Meyer, Analilia Arroyo-Becerra

**Affiliations:** 1 Laboratorio de Genómica Funcional y Biotecnología de Plantas, Centro de Investigación en Biotecnología Aplicada, Instituto Politécnico Nacional (CIBA-IPN), Tepetitla de Lardizábal, Tlaxcala, México; 2 Departamento de Ingeniería Genética, Centro de Investigación y de Estudios Avanzados (CINVESTAV Unidad Irapuato), Irapuato, Guanajuato, México; 3 Consejo Nacional de Ciencia y Tecnología, Instituto Politécnico Nacional-Centro de Investigación en Biotecnología Aplicada (CIBA-IPN), Ciudad de México, México; 4 Departamento de Biología Molecular de Plantas, Instituto de Biotecnología, Universidad Nacional Autónoma de México (IBT-UNAM), Cuernavaca, Morelos, México; 5 Departamento de Biotecnología Agrícola, Centro Interdisciplinario de Investigación para el Desarrollo Integral Regional, Instituto Politécnico Nacional (CIIDIR-IPN Unidad Sinaloa), Guasave, Sinaloa, México; United Arab Emirates University, UNITED ARAB EMIRATES

## Abstract

Sugars act not only as substrates for plant metabolism, but also have a pivotal role in signaling pathways. Glucose signaling has been widely studied in the vascular plant *Arabidopsis thaliana*, but it has remained unexplored in non-vascular species such as *Physcomitrella patens*. To investigate *P*. *patens* response to high glucose treatment, we explored the dynamic changes in metabolism and protein population by applying a metabolomic fingerprint analysis (DIESI-MS), carbohydrate and chlorophyll quantification, Fv/Fm determination and label-free untargeted proteomics. Glucose feeding causes specific changes in *P*. *patens* metabolomic fingerprint, carbohydrate contents and protein accumulation, which is clearly different from those of osmotically induced responses. The maximal rate of PSII was not affected although chlorophyll decreased in both treatments. The biological process, cellular component, and molecular function gene ontology (GO) classifications of the differentially expressed proteins indicate the translation process is the most represented category in response to glucose, followed by photosynthesis, cellular response to oxidative stress and protein refolding. Importantly, although several proteins have high fold changes, these proteins have no predicted identity. The most significant discovery of our study at the proteome level is that high glucose increase abundance of proteins related to the translation process, which was not previously evidenced in non-vascular plants, indicating that regulation by glucose at the translational level is a partially conserved response in both plant lineages. To our knowledge, this is the first time that metabolome fingerprint and proteomic analyses are performed after a high sugar treatment in non-vascular plants. These findings unravel evolutionarily shared and differential responses between vascular and non-vascular plants.

## Introduction

Both microorganisms and multicellular organisms coordinate their metabolic activity according to changes in nutrient availability. This coordination is achieved through the sensing of energy availability and relaying this information to metabolic regulators that ultimately impact their growth and development [1, 2]. In plants, sensing the availability of energy in the form of sugars is particularly critical since these molecules play a key role in the carbohydrate metabolism and cellular redox balance through their close rapport with fatty acid β-oxidation, respiration, and photosynthesis [3–6].

In vascular plants, various forms of sugar have emerged as important regulators of plant development, glucose is the most prominent and evolutionarily conserved [4]. In the last decades, extensive studies in *A*. *thaliana* have revealed that sugars have dual function acting as a fuel and also as signaling molecules. Both functions play pivotal roles in integrating the metabolic, developmental, and environmental cues required for plant survival [4]. In *Arabidopsis*, multiple signals that modulate the growth and development have been described, this process requires energy and functional ribosomes, in this sense sugars supply energy and carbon building blocks for protein and RNA biosynthesis [6–8]. Forward genetics, involving the screening of mutants insensitive or hypersensitive to the effects of glucose on *Arabidopsis* seedling development, has been a powerful approach for identifying genes involved in glucose sensing and signaling [9–13]. Interestingly, these screenings have identified mutants associated with abscisic acid (ABA), ethylene, auxin, cytokinin, stringolactones, gibberellins, and brassinosteroids, thus demonstrating an active cross-talk between sugar and phytohormone responses [4, 6, 7, 12, 14, 15]. One of the key components of glucose sensing and signaling is hexokinase1 (HXK1), an evolutionary conserved glycolytic enzyme responsible for regulating the expression of a broad range of genes, in addition to its standard catalytic function [6, 10, 16–19]. In addition, glucose activates TOR (Target of rapamycin) complex, which has a crucial role as an energy master regulator of plant growth, development, root meristem activity, cell cycle control, flowering, senescence through the modulation of transcription, ribosome biogenesis and translation [6–8]. In plants two systems that respond to changes in nutrient and energy status have been reported, the TOR complex kinase, which promotes growth in response to high glucose [20], and the plant Snf1-related kinase 1 (SnRK1) which is active upon sugar deprivation [21] TOR and SnRK1 act downstream of sugar sensing and their activities are modulated by the sugar status of plants [8].

Genome-wide expression profiling studies have revealed that high glucose concentrations alter the expression of genes involved in metabolic processes, signal transduction, metabolite transport, and stress responses [10, 18, 22–24]. Other important processes regulated by sugar include post-transcriptional level regulation that comprises transcript stability and processing, synthesis of proteins regulating selective mRNA translation, ribosome biosynthesis, protein stability/degradation, and modulation of enzymatic activities [7, 8, 13]. Examining glucose-mediated changes at the transcriptional level is informative, but the proteins are ultimately responsible for nearly every task of cellular activity and metabolism. Glucose sensing and signaling through the mentioned pathways link carbon nutrient status to plant growth and development, and several aspects of sugar perception and signaling are likely to be unique to higher plants [25]. Then, some of these mechanisms could be conserved in ancestral lineages of plants, such as bryophytes, even though the information available about these mechanisms is scarce. In this scenario, exploring the role of glucose as a signaling molecule in non-vascular plants is important and necessary.

Vascular plants (which include xylem and phloem tissues to transport water, nutrients, phytohormones, and photosynthates) have been used as model plants to study several aspects of physiology, molecular biology, and development [26]. However, some aspects such as performing crosses to obtain stable phenotypes, the presence of multiple cell types and tissues, leading to complex sink/source relations that limit some studies. In that sense, *P*. *patens* is a bryophyte lacking the vascular system (thereby it requires a constant co-equilibration of tissue water content with the environment) represents a less complex plant [27, 28]. This moss has been a premier model system as it possesses a simple anatomy and developmental pattern, a short life cycle, a haploid genome during most of its life cycle, a high rate of homologous recombination, allowing the study of the biology and evolution of non-vascular plants, also *P*. *patens* was the first non-seed plant to have its genome sequenced [27, 29, 30]. The evolutionary importance of *P*. *patens* is highlighted since it is phylogenetically related to the first plants that conquered the earth. Similar to the first terrestrial plants, *P*. *patens* had to acquire mechanisms of tolerance to grow under demanding environmental conditions, including salinity, cold, and drought [31–33]. In *P*. *patens*, exogenous glucose stimulates caulonemal filament formation, a response that is lost in an *hxk1* knockout mutant [34, 35]. Since caulonemal formation is the first step towards the production of gametophores, high-energy availability seems to be the signal for sexual reproduction. Some efforts have been made to study the mechanisms by which *P*. *patens* responds to glucose stimulus, but until now, conclusive results have been elusive [34, 35].

Holistic changes at proteome and metabolome levels are inherent for adaptation to any physiological condition. To discover the role of glucose in *P*. *patens*, a comprehensive approach was used to determine the dynamic changes in the metabolism and protein population after glucose exposure. High glucose conditions gave rise to a glucose-specific osmotic-independent perturbation in metabolomic fingerprints, carbohydrate content and metabolism; specifically, the number of certain proteins related to translation, photosynthesis, oxidative stress, and protein refolding. To our knowledge, this is the first time that metabolome fingerprint and proteomic analyses are performed in a non-vascular plant after a high sugar concentration treatment. Our findings contribute to unravel differential, as well as the overlapping responses to glucose between *A*. *thaliana* and *P*. *patens*, two model plants representing evolutionarily distant plant lineages, expanding the knowledge about the role of glucose as a specific signaling molecule in non-vascular plants.

## Materials and methods

### Plant material and growth conditions

Protonemata of *P*. *patens* ecotype Gransden were grown in PpNH_4_ medium that containing 0.68 mM MgSO_4_•7H_2_O, 1.836 mM KH_2_PO_4_, 2.452 mM CaNO_3_•4H_2_O, 2.714 mM Di-ammonium tartrate, 6.18x10^-4^ mM FeSO_4_•7H_2_O, microelements (9.9nM H_3_BO_3_, 1.6 nM CuSO_4_·5H_2_O, 1.4 nM MnCl_2_·4H_2_O, 1.5 nM CoCl_2_·6H_2_O, 1.3 nM ZnSO_4_·7H_2_O, 1.6 nM KI, 0.8 nM Na_2_MoO_4_·2H_2_) and agar (7g/L). For all treatments, culture plates were maintained under standard conditions in a growth room at 23±1°C under a 16/8 h light/dark photoperiod with a light intensity of 55 μmol photons m^-2^s^-1^. To evaluate glucose effects, 10-day old protonemata were exposed to 0 and 300 mM of glucose for 24 h (a complete circadian cycle to avoid circadian rhythms effects). Plants grown in medium with no glucose was considered the control condition. Additionally, sorbitol was used as an osmotic control [32, 36, 37]. Three independent biological experiments were performed. Protonemata from the same experiment but independent samples were used for metabolomic fingerprint, carbohydrate quantification, and proteomic analysis. Samples from an independent experiment were used for chlorophyll a and b quantification. The protonemal tissues to measure the Fv/Fm were from another independent experiment. Three independent replicates were used for all experiments.

### Direct-injection electrospray ionization mass spectrometry

A direct-injection electrospray ionization−mass spectrometry (DIESI−MS) assay was performed in a DIESI–MS employing an SQD2 with a quadrupole analyzer (Waters) and MassLynx 4.0 as described previously [38, 39] for each one of the independent biological replicates. This strategy allows the detection of significant differences among MS profiles and the collection of large amounts of quantitative metabolic data. Therefore, the rapidity of the analyses permits “bed-side” monitoring of plants physiological states.

### Carbohydrate quantification

Carbohydrate quantification was performed on protonemata exposed to either glucose or sorbitol treatments. Tissues were frozen with liquid nitrogen and lyophilized followed by extraction, and glucose, fructose, sucrose, and starch contents were measured as previously reported [40].

### Protein extraction

The protonemata exposed to 0 and 300 mM of glucose or sorbitol for 24 h were frozen in liquid nitrogen. Plant tissue was ground to a fine powder in liquid nitrogen and homogenized on ice for 1 h with 500 uL of ice-cold extraction buffer (8 M urea, 2 M thiourea, 0.04 mM dithiothreitol) supplemented with a cocktail of protease and phosphatase inhibitors (Roche Diagnostics). After centrifugation at 4°C for 20 min, the supernatant was collected and precipitated overnight with acetone at –20°C. The pellet was washed with cold 90% (v/v) acetone and suspended in ABC buffer (100 mM ammonium bicarbonate, 2% SDS w/v). Total protein was determined with the Bradford method. Protein quality and quantity were verified by SDS-PAGE.

### Tryptic digestion and LC-MS analysis

Total proteins from three biological replicates were reduced with dithiothreitol (DTT), alkylated with iodoacetamide (Sigma Aldrich), and digested with trypsin (Promega Modified Trypsin Sequencing Grade). The resulting peptides were applied to a pump LC-MS nanoflow EASY-nLC II instrument coupled to a mass spectrometer LTQ Orbitrap-Velos system with nano-electrospray ionization (Thermo Fisher Scientific Co., San Jose, CA). To validate MS/MS-based peptide and protein identifications, algorithms, and tools were used as previously reported [41] and are described in the following sections.

### Criteria for protein identification

All MS/MS samples from three biological replicates were analyzed using Sequest (https://omictools.com/sequest-tool) and X! Tandem (http://wiki.thegpm.org/wiki/X!!Tandem) for peptide identification. Both tools were set up to search on the uniprot-physcomitrella+patens.fasta file (UP000006727, 35539 entries) assuming trypsin digestion. Sequest and X! Tandem were used considering a fragment ion mass tolerance of 0.60 Da and a parent ion tolerance of 20 ppm. Cysteine carbamidomethyl was considered as a fixed modification, whereas histidine carbamidomethyl, methionine oxidation, and Glu->pyro-Glu, Gln->pyro-Glu and ammonia-loss of the N-terminus were specified as variable modifications. Protein identification from the three biological replicates was carried out using the software tool Scaffold (version Scaffold_4.4.6, Proteome Software Inc., Portland, OR). Accordingly, peptide identifications were accepted if they could be established at greater than 96.0% probability by the Scaffold Local FDR algorithm. Protein identifications were accepted if they could be established at greater than 7.0% probability to achieve an FDR <1.0% and contained at least two identified peptides. Protein probabilities were assigned with the Protein Prophet algorithm [42]. Proteins that contained similar peptides and could not be differentiated based on the MS/MS analysis alone were grouped to satisfy parsimony principles. Proteins sharing significant peptide similarities were grouped into clusters. Proteins were annotated with gene ontology (GO) terms from gene association.goa [43]. Raw data is available at https://data.mendeley.com/datasets/t5m28m66vc/1 (doi: 10.17632/t5m28m66vc.1).

### Measurement of chlorophyll fluorescence

The maximal rate of PSII was determined by variable fluorescence (Fv)/maximal fluorescence (Fm) measurements. Briefly, 10-day old protonemata were exposed to 0 and 300 mM of either glucose or sorbitol for 24 h. Dark adaptation for 10 to 15 min was allowed before each measurement. Then Fv/Fm was measured using the fluorimeter FluorPen100 (Photon Systems Instruments, Czech Republic).

### Chlorophyll extraction and quantification

Chlorophyll was extracted with 80% acetone from lyophilized protonemata previously exposed to either glucose or sorbitol. The optical density (absorbance) of the extract was measured with a microplate reader (Epoch microplate spectrophotometer, BioTek). Light absorbance was measured at 663 and 645 nm wavelengths (maximum absorption of chlorophyll a and b). Chlorophyll concentrations were then calculated according to Wellburn [44] and expressed as mg chlorophyll per g dry weight (mg/g DW).

### Additional bioinformatics tools

Proteins were classified into cellular components according to GO annotations based on the UniProt database (http://www.uniprot.org/). Functional protein association networks of specific glucose-responsive proteins were performed with the STRING tool (https://string-db.org) [45]. The interactions between proteins were visualized in Cytoscape software (version 3.6.1[http://cytoscape.org]) [46]. GO enrichment analysis of the clusters obtained was performed using the Blast2GO software (version 5.2.4) [47], Cytoscape plugin, ClueGO (version 2.5.2; Laboratory of Integrative Cancer Immunology) [48] and KEGG pathway maps (Kyoto Encyclopedia of Genes and Genomes, Kanehisa Laboratories) (data not shown).

### Statistical analyses

The statistical analysis for DIESI-MS was made as previously reported [38, 39]. For carbohydrate quantification, measurement of chlorophyll fluorescence and chlorophyll quantification, three independent samples for each treatment were measured and verified by three technical replicates. Analysis of variance (ANOVA) was done; different letters indicate statistically significant differences (*P* ≤0.05) using a post hoc Tukey test (SAS university edition). In the case of the proteomic analysis, the Scaffold Quantitative Testing was used for fold change and statistical calculation based on spectrum counting. The proteins with differential expression were selected using a T-test with *P*≤0.05 and Hochberg-Benjamini correction (α = 0.00031). The fold changes were calculated based on the relative protein abundance found in the treatment groups with respect to those identified in the control groups.

## Results

### Glucose alters moss metabolism independently from an osmotic response

**Fig 1:**
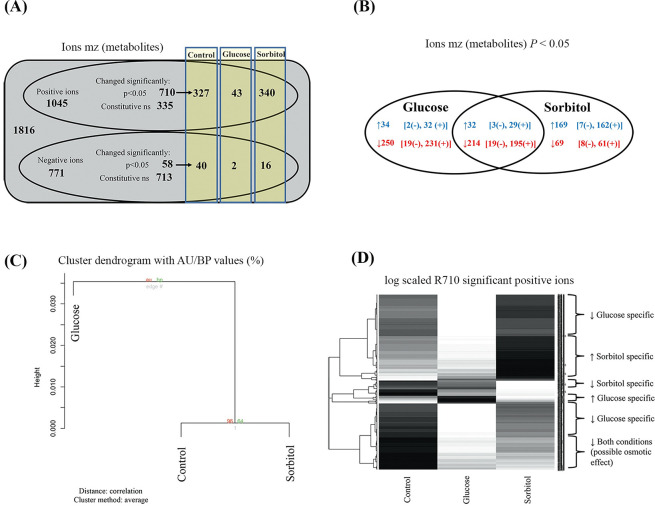
*P*. *patens* metabolomic fingerprinting in response to glucose and sorbitol. Protonemata were exposed to 0 mM (control condition) and 300 mM of either glucose or sorbitol for 24 h. (A) Diagram representing the number of positive and negative ions identified under the evaluated conditions (ns, non-significant). (B) Venn diagram showing the distribution of the 710 positive (+) and 58 negative (-) ions that increased (blue) and decreased (red) in response to glucose and sorbitol treatments. (C) Cluster dendrogram showing the metabolomic fingerprint indicating a glucose specific response. (D) Heatmap profile showing clustering based on correlation R applied to positive ions. The metabolomic fingerprint is represented as a grayscale barcode and the ion similarity is revealed by the left dendrogram. The grayscale depicted the relative intensity (ion abundance) under the different conditions (black indicates high, and white indicates low). Results correspond to three independent biological samples.

In order to understand the total effect on *P*. *patens* tissues that were exposed to high glucose concentration (300 mM) for 24 h, we first performed mass spectrometry fingerprinting with DIESI-MS [39].To distinguish between glucose-specific and osmotic responses, sorbitol was included as a control in our experimental design. Through DIESI-MS analysis, a total of 1816 mass peaks were identified (1045 positively charged ions and 771 negatively charged ions). Using a significance of *P<*0.05, 710 positive ions showed changes from which 327 correspond to the control conditions, 43 to glucose treatment and 340 to sorbitol treatment. Regarding the negative ions, 58 showed significant changes (*P*< 0.05), 40 ions under control condition, two in response to glucose and 16 in sorbitol treatment (Fig 1A and S1 Table). The distribution of the increased and decreased 710 positive ions and 58 negative ions is shown in Fig 1B. Compared to control conditions, both glucose and sorbitol feeding led to an increase of 32 ions and a decrease of 214 ions (Fig 1B and S1 Fig). Glucose led to a specific increase in 34 ions (32 positives and two negatives) and a specific decrease in 250 ions (231 positives and 19 negatives), whereas sorbitol feeding caused a specific increase in 169 ions (162 positives and seven negatives) and a decrease in 69 ions (61 positives and eitght negatives) (Fig 1B, S1 Fig and S1 Table). Shared glucose and sorbitol condition responses could be interpreted as an osmotic effect associated response (Fig 1B and S1 Fig). The metabolomic fingerprint revealed a dendrogram in which sorbitol and control clustered together, whereas glucose led to a separate branch (Fig 1C). Overall, the metabolomic fingerprint changed in the presence of 300 mM glucose and was significantly different from sorbitol and control conditions, indicating a glucose-specific response (Fig 1 and S1 Table). In addition to the shared response, significant metabolomic differences distinguished the samples. Using statistical data mining with *P* <0.05, a comprehensive list of ions was obtained and then grouped into categories with their respective mass charge ratio (mz value) (S1 Fig). Several heatmap-bicluster figures were constructed, selecting the significant negative ions only (S2 Fig), all the significant positive ions (Fig 1D), or only the most intense significant positive ions (S3 Fig). In all cases, an optimized hierarchical clustering based on correlation as previously described was applied [49]. Those grayscale heatmaps depicted the relative intensity (ion abundance) under the different conditions (black indicates high, and white indicates low, as shown in Fig 1D and S2 and S3 Figs). Hierarchical clustering grouped the glucose- or sorbitol-specific ions (S1–S3 Figs). Glucose feeding led to a preferential decrease of a larger number of metabolites, whereas sorbitol feeding led to a decrease of a fewer number of ions (Fig 1B and S1 Fig).

**Fig 2:**
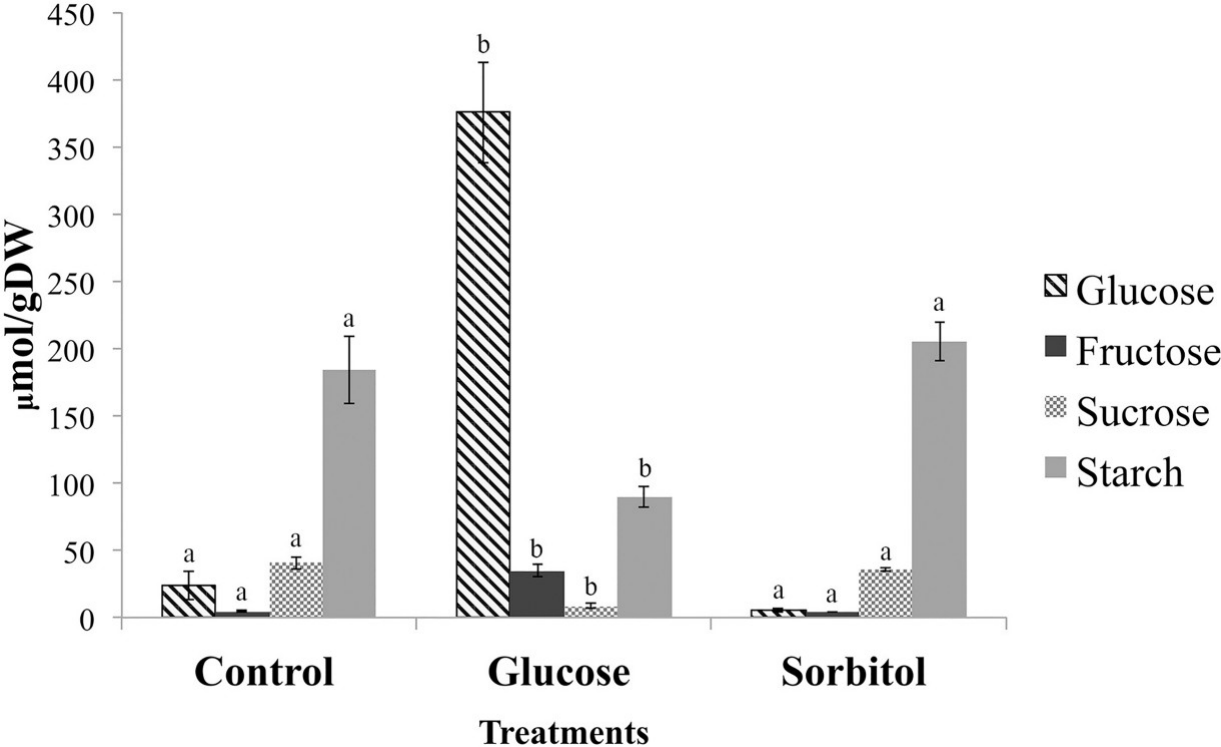
Carbohydrate content in *P*. *patens* exposed to glucose and sorbitol. Hexoses (such as glucose and fructose) in addition to sucrose and starch levels were measured upon treatments of protonemata with or without 300 mM of either glucose or sorbitol for 24 h. Graphical representation of mean ± SE of three independent biological samples. An analysis of variance (ANOVA) was done, and different letters indicate statistically significant differences (*P* ≤0.05) using a post hoc Tukey test (SAS university edition).

To examine some of the biochemical adjustments caused by the treatments, the levels of four main carbohydrates (glucose, fructose, sucrose, and starch) were determined under the same experimental conditions. As expected, glucose feeding led to a strong increase in the hexoses, glucose and fructose (Fig 2). In contrast, the 300 mM sorbitol feeding caused a marginal decrease in internal glucose levels but did not alter the fructose, sucrose, or starch pools compared to control conditions (Fig 2). Unexpectedly, glucose feeding despite increasing available hexose levels led to a sucrose and starch decrease (Fig 2). Altogether these observations highlight specific and differential effects of glucose compared to sorbitol in *P*. *patens*, therefore supporting our metabolomic fingerprint analysis results (Fig 1; S2 and S3 Figs and S1 Table). Altogether, the non-biased metabolomic fingerprinting approach and the targeted carbohydrate assay confirmed that glucose caused a specific response that was independent of its osmotic effect. The fact that not all metabolites increased after glucose feeding pointed to a coordinated response of several enzymes within the metabolic network. Considering the great difficulty in measuring flux and catalytic activities of a large number of unknown enzymes, a quantitative proteomic approach was then pursued to detect more or less abundant proteins under these conditions.

### Proteomic analysis in response to glucose

To gain insights into the biological processes responsible for the observed metabolic changes in response to glucose signals, we performed a label-free untargeted proteomic method to establish the proteins altered by high glucose, as well as sorbitol treatments, compared to the control condition. A total of 319 proteins in 212 clusters were reliably identified (S2 Table). According to our established discrimination criteria (see Materials and Methods), 240 proteins were classified as constitutive, whereas 79 showed differential expression in high glucose (53 proteins) and sorbitol (26 proteins) treatments in comparison to the control. From the 53 proteins differentially expressed in response to glucose, 44 proteins were more abundant (increased significantly), while 9 proteins were less abundant (decreased significantly) under these treatment conditions (Table 1). Regarding the 26 differential proteins corresponding to the osmotic control treatment using sorbitol, only one protein increased and 25 decreased (Table 2). Interestingly, six proteins: two Phosphoribulokinase, one UTP-glucose-1-phosphate uridylyltransferase, one Fasciclin-like protein, and two predicted proteins were identified as common to glucose and sorbitol treatments (Tables 1 and 2). In summary, the number of differential proteins identified between glucose and sorbitol treatments supports our previous observation at the metabolic level, namely that the molecular glucose-induced responses are specific and clearly distinguishable from its osmotic effects.

**Table 1:** Biological processes classification of the 53 Up- and Down-regulated proteins in response to glucose treatment in *P*. *patens*.

Treatment	UniProt ID	STRING ID	Description	Subcellular localization	Fold change (log_2_)
**Glucose (Up 44 proteins)**	**Translation [GO:0006412]**				
	A9RI50_PHYPA	PP1S10_102V6.1	Predicted protein	Cytosolic large ribosomal subunit [GO:0022625]	12.971272
	A9RN38_PHYPA	PP1S18_113V6.1	Predicted protein	Cytosolic large ribosomal subunit [GO:0022625]	12.860161
	A9RMS0_PHYPA	PP1S17_306V6.1	40S ribosomal protein S3a	Cytosolic small ribosomal subunit [GO:0022627]	12.249915
	A9TAH6_PHYPA	PP1S194_130V6.1	40S ribosomal protein S12	Cytosolic small ribosomal subunit [GO:0022627]	2.6954634
	A9RT00_PHYPA	PP1S26_289V6.1	40S ribosomal protein S12	Cytosolic small ribosomal subunit [GO:0022627]	2.5598142
	A9SXV6_PHYPA	PP1S134_153V6.1	Predicted protein	Large ribosomal subunit [GO:0015934]	2.2011025
	A9RKD8_PHYPA	PP1S14_191V6.1	Predicted protein	Large ribosomal subunit [GO:0015934]	0.79837275
	A9SH83_PHYPA	PP1S78_212V6.1	Predicted protein	Large ribosomal subunit [GO:0015934]	0.67553025
	**Translational elongation [GO:0006414]**				
	A9T682_PHYPA	PP1S172_22V6.1	EF1B gamma class glutathione S-transferase	-	2.4289958
	**Photosynthesis [GO:0015979]**				
	A9SL09_PHYPA	PP1S89_62V6.1	PsaH photosystem I reaction center subunit	Chloroplast thylakoid membrane [GO:0009535]; photosystem I reaction center [GO:0009538]	4.261696
	A9TCU9_PHYPA	PP1S206_11V6.1	Predicted protein	Chloroplast thylakoid membrane [GO:0009535]; photosystem I reaction center [GO:0009538]	3.8950694
	A9TU20_PHYPA	PP1S319_36V6.1	Predicted protein	Photosystem I reaction center [GO:0009538]	1.5972413
	A9SRS0_PHYPA	PP1S109_145V6.1	Ribulose bisphosphate carboxylase small chain	Plastid [GO:0009536]	0.5064318
	A9S3R8_PHYPA	PP1S46_42V6.1	Ribulose bisphosphate carboxylase small chain	Plastid [GO:0009536]	0.5776918
	**Electron transport chain [GO:0022900]**				
	A9RDX6_PHYPA	PP1S3_520V6.1	Plastocyanin	Chloroplast thylakoid membrane [GO:0009535]	13.087763
	Q9SXW9_PHYPA	PP1S254_25V6.1	Plastocyanin, chloroplastic	Chloroplast thylakoid membrane [GO:0009535]	12.194749
	**Tetrapyrrole biosynthetic process [GO:0033014]**				
	A9S7G9_PHYPA	PP1S54_66V6.4	Predicted protein	Chloroplast [GO:0009507]	1.6633108
	**Cellular response to oxidative stress [GO:0034599]**				
	A9RW02_PHYPA	PP1S31_128V6.1	Peroxiredoxin	Mitochondrion [GO:0005739]; cytoplasm [GO:0005737]	12.149561
	A9SX65_PHYPA	PP1S131_153V6.1	Superoxide dismutase [Cu-Zn]	Cytoplasm [GO:0005737]; extracellular space [GO:0005615]	3.751367
	A9SX31_PHYPA	PP1S131_71V6.4	Superoxide dismutase [Cu-Zn]	Cytoplasm [GO:0005737]; extracellular space [GO:0005615]	3.0703168
	**Oxidation-reduction process [GO:0055114]**				
	Q2I826_PHYPA	PP1S237_59V6.5	Monodehydroascorbate reductase III	-	13.932129
	**Protein refolding [GO:0042026];**				
	A9TK88_PHYPA	PP1S249_62V6.1	Peptidyl-prolyl cis-trans isomerase (PPIase)	Chloroplast [GO:0009507]; cytosol [GO:0005829]; golgi apparatus [GO:0005794]; plasma membrane [GO:0005886]	14.075424
	A9ST56_PHYPA	PP1S115_168V6.2	Predicted protein	Chloroplast [GO:0009507]	11.758743
	A9T8E8_PHYPA	PP1S183_47V6.1	Predicted protein	Cytoplasm [GO:0005737]; mitochondrion [GO:0005739]; vacuolar membrane [GO:0005774]	8.869213
	**Cellular response to heat [GO:0034605]**				
	A9TQG3_PHYPA	PP1S288_23V6.1	Predicted protein	Cytoplasm [GO:0005737]; endoplasmic reticulum chaperone complex [GO:0034663]; endoplasmic reticulum lumen [GO:0005788]; membrane [GO:0016020]; nucleus [GO:0005634]	13.662725
	**Fatty acid biosynthetic process [GO:0006633]**				
	A9RMZ3_PHYPA	PP1S18_23V6.1	Biotin carboxylase	-	3.6311908
	A9TC15_PHYPA	PP1S201_89V6.1	Predicted protein	-	2.1733608
	**ATP synthesis coupled proton transport [GO:0015986]**				
	A9RHZ0_PHYPA	PP1S10_393V6.1	Predicted protein	Membrane [GO:0016020]	11.004703
	A9SYE0_PHYPA	PP1S137_86V6.1	Predicted protein	Membrane [GO:0016020]	10.605091
	**Glutamate catabolic process [GO:0006538]**				
	A9RXP9_PHYPA	PP1S34_308V6.2	Glutamate decarboxylase	Cytosol [GO:0005829]	13.399181
	**Glucose metabolic process [GO:0006006]**				
	A9RDK9_PHYPA	PP1S3_238V6.4	Glyceraldehyde-3-phosphate dehydrogenase	Cytosol [GO:0005829]	13.15653
	**S-adenosylmethionine biosynthetic process [GO:0006556]**				
	A9SRR7_PHYPA	PP1S244_65V6.2	Predicted protein	Cytosol [GO:0005829]	11.045008
	**Biosynthetic process [GO:0009058]**				
	A9TEP5_PHYPA	PP1S215_28V6.1	Predicted protein	-	3.6568143
	**Glycine decarboxylation [GO:0019464]**				
	A9TNF2_PHYPA°	(Without STRING ID)	Glycine cleavage system H protein	Mitochondrion [GO:0005739]	1.0921887
	**Cell wall modification [GO:0042545]**				
	A9TEQ0_PHYPA	PP1S215_36V6.1	Pectinesterase	Cell wall [GO:0005618]	0.6753476
	**Without GO associated**				
	A9SUK7_PHYPA	PP1S120_139V6.3	Predicted protein	-	12.738213
	A9SVT2_PHYPA	PP1S126_26V6.2	Predicted protein	Cytoplasm [GO:0005737]	12.63903
	A9RBY5_PHYPA	PP1S1_765V6.1	Uncharacterized protein	Chloroplast thylakoid membrane [GO:0009535]; integral component of membrane [GO:0016021]	12.469591
	A9TVV6_PHYPA	PP1S339_37V6.1	Predicted protein	-	4.1429434
	A9RHV4_PHYPA	PP1S10_319V6.1	Predicted protein	Nascent polypeptide-associated complex [GO:0005854]	3.14782
	A9SV00_PHYPA	PP1S122_100V6.1	Predicted protein	Nascent polypeptide-associated complex [GO:0005854]	2.9994178
	A9U4U1_PHYPA	PP1S539_1V6.1	Predicted protein	Nascent polypeptide-associated complex [GO:0005854]	2.9973712
	A9TWS3_PHYPA	PP1S351_30V6.1	Dihydrolipoamide acetyltransferase component of pyruvate dehydrogenase complex	-	2.350117
	A9RWX8_PHYPA	PP1S33_209V6.1	Predicted protein	Cytoplasm [GO:0005737]	1.543775
**Glucose (Down 9 proteins)**	**Carbohydrate metabolic process [GO:0005975]**				
	A9TRN4_PHYPA*	PP1S299_3V6.1	Phosphoribulokinase	Chloroplast [GO:0009507]	-1.5467525
	A9SXF3_PHYPA*	PP1S132_175V6.1	Phosphoribulokinase	Chloroplast [GO:0009507]	-1.2861613
	A9SF03_PHYPA*	PP1S72_25V6.1	Predicted protein	Cytosol [GO:0005829]	-0.5654774
	A9TPV2_PHYPA*	PP1S283_22V6.2	UTP—glucose-1-phosphate uridylyltransferase	Cytoplasm [GO:0005737]	-0.49702644
	**Oxidation-reduction process [GO:0055114]**				
	A9RJ44_PHYPA	PP1S12_209V6.2	Predicted protein	-	-1.3040282
	**Translational elongation [GO:0006414]**				
	A9T0S0_PHYPA	PP1S147_106V6.1	Elongation factor Tu	Mitochondrion [GO:0005739]	-0.7298387
	**Methionine biosynthetic process [GO:0009086]**				
	A9RWS2_PHYPA	PP1S33_110V6.2	Predicted protein	-	-0.7030835
	**Without GO associated**				
	Q4A3V1_PHYPA*	PP1S545_14V6.1	Fasciclin-like protein	Extracellular space [GO:0005615]	-0.8814069
	A9TIB8_PHYPA*	PP1S237_14V6.1	Predicted protein	-	-0.3836249

Note: ° Glucose-induced protein found less abundant under sorbitol treatment.

* Common proteins found less abundant under sorbitol treatment.

**Table 2:** Biological processes classification of the 26 Up- and Down-regulated proteins in response to sorbitol treatment in *P*. *patens*.

Treatment	UniProt ID	STRING ID	Description	Subcellular localization	Fold change (log_2_)
**Sorbitol (Up 1 protein)**	**Without GO associated**				
	A9U4I0_PHYPA	PP1S517_11V6.2	Predicted protein	-	13.096634
**Sorbitol (Down 25 proteins)**	**Carbohydrate metabolic process [GO:0005975]**				
	A9SXF3_PHYPA*	PP1S132_175V6.1	Phosphoribulokinase	Chloroplast [GO:0009507]	-13.6788845
	A9U222_PHYPA	PP1S429_29V6.1	Predicted protein	Cytoplasm [GO:0005737]	-2.6723442
	A9TRN4_PHYPA*	PP1S299_3V6.1	Phosphoribulokinase	Chloroplast [GO:0009507]	-2.6158485
	A9S1S8_PHYPA	PP1S41_162V6.1	Predicted protein	Cytoplasm [GO:0005737]	-2.3215294
	A9TPV2_PHYPA*	PP1S283_22V6.2	UTP—glucose-1-phosphate uridylyltransferase	Cytoplasm [GO:0005737]	-1.9669869
	A9S087_PHYPA	PP1S39_82V6.1	UTP—glucose-1-phosphate uridylyltransferase	Cytoplasm [GO:0005737]	-1.9669869
	A9SF03_PHYPA*	PP1S72_25V6.1	Predicted protein	Cytosol [GO:0005829]	-1.7638044
	**Proton trans-membrane transport [GO:1902600]**				
	A9TYF3_PHYPA	PP1S372_16V6.1	Predicted protein	Chloroplast thylakoid membrane [GO:0009535]	-2.22756
	A9U2Q2_PHYPA	PP1S445_15V6.1	Predicted protein	Integral component of membrane [GO:0016021]; membrane [GO:0016020]	-1.6320391
	A9TWH1_PHYPA	PP1S346_35V6.1	Predicted protein	Integral component of membrane [GO:0016021]	-0.8361692
	**Cell redox homeostasis [GO:0045454])**				
	A9SNH9_PHYPA	PP1S98_132V6.1	Dihydrolipoyl dehydrogenase	Cell [GO:0005623]	-13.520764
	**Protein glutathionylation [GO:0010731]**				
	A9RJE6_PHYPA	PP1S12_401V6.1	Predicted protein	-	-1.7032927
	**Glycine biosynthetic process [GO:0019265]**				
	A9RNQ2_PHYPA	PP1S19_25V6.1	Predicted protein	Peroxisome [GO:0005777]	-2.4561348
	A9TY57_PHYPA	PP1S369_6V6.1	Predicted protein	Peroxisome [GO:0005777]	-2.4561348
	**Protein refolding [GO:0042026]**				
	A9SLL3_PHYPA	PP1S91_109V6.1	Predicted protein	Cytoplasm [GO:0005737]	-2.2907598
	A9SNF1_PHYPA	PP1S97_279V6.1	Uncharacterized protein	Cytoplasm [GO:0005737]	-0.4218207
	**Microtubule-based process [GO:0007017]**				
	Q8H932_PHYPA	(Without STRING ID)	Tubulin alpha chain	Cytoplasm [GO:0005737]; microtubule [GO:0005874]	-3.0423586
	**Translation [GO:0006412]**				
	A9TG34_PHYPA	PP1S223_73V6.1	40S ribosomal protein S8	Cytosolic small ribosomal subunit [GO:0022627]	-2.6163216
	**Photosynthesis [GO:0015979]**				
	A9SGR0_PHYPA	PP1S77_69V6.1	Predicted protein	Photosystem I reaction center [GO:0009538]	-1.562467
	**Glycine decarboxylation [GO:0019464]**				
	A9TNF2_PHYPA°	(Without STRING ID)	Glycine cleavage system H protein	Mitochondrion [GO:0005739]	-0.77886844
	**Without GO associated**				
	A9TIB8_PHYPA*	PP1S237_14V6.1	Predicted protein	-	-12.283276
	A9TBG2_PHYPA	PP1S198_154V6.3	Actin 7	Cytoskeleton [GO:0005856]	-2.5983582
	A9SYJ1_PHYPA	PP1S137_232V6.1	Predicted protein	-	-2.0514417
	A9SYH4_PHYPA	PP1S137_194V6.1	Predicted protein	-	-2.0336146
	Q4A3V1_PHYPA*	PP1S545_14V6.1	Fasciclin-like protein	Extracellular space [GO:0005615]	-1.7911283

Note: °Common protein found increased (more abundant) in response to glucose.

*Common proteins found decreased (less abundant) in response to glucose.

### Glucose induces proteins mainly related to translation, photosynthesis, cellular response to oxidative stress and protein refolding

**Fig 3:**
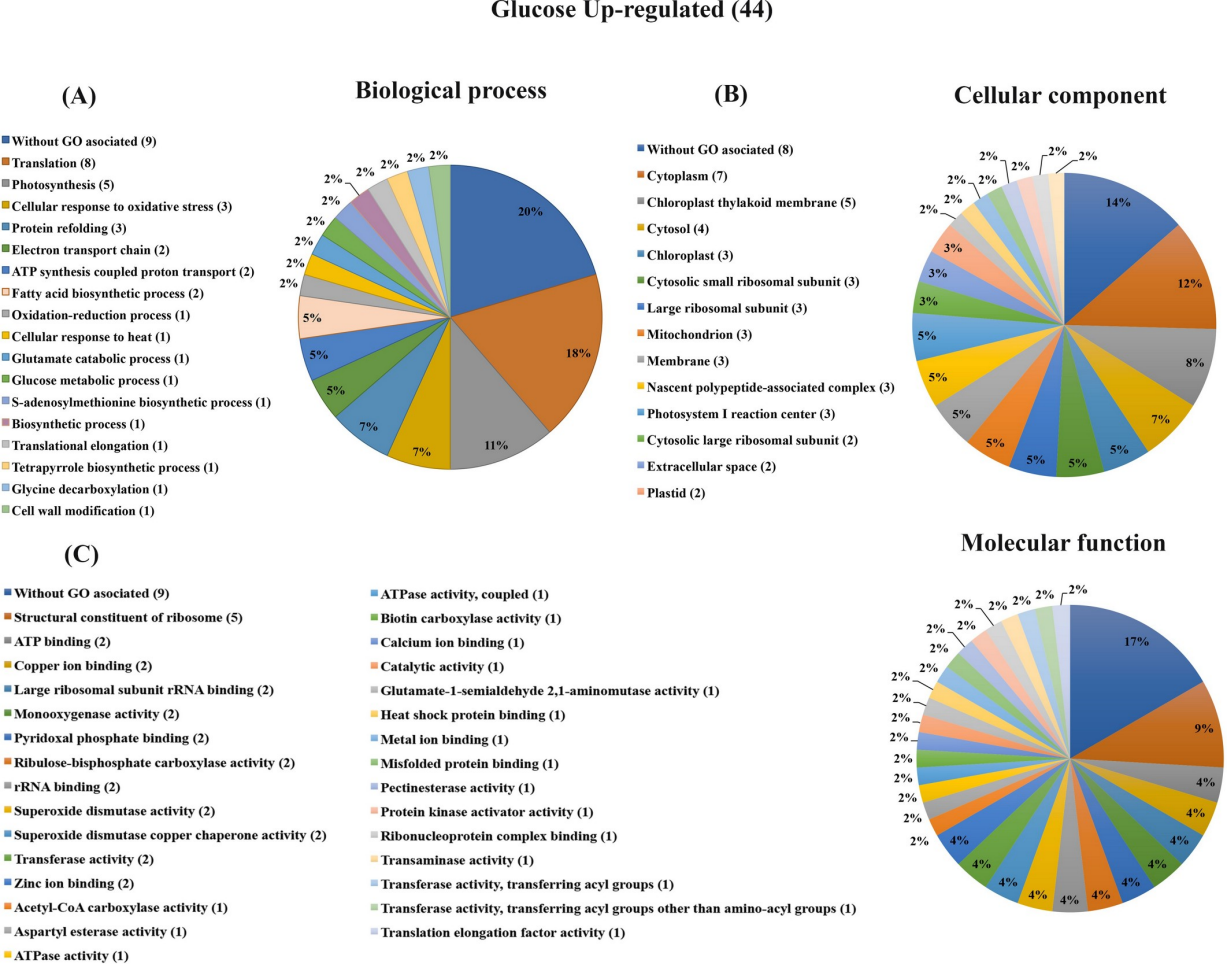
Graphical representation of gene ontology classification for proteins up-regulated by glucose treatment. (A) Biological process classification. Proteins involved in translation, photosynthesis, cellular responses to oxidative stress, and protein refolding were predominant. (B) Cellular component. The majority of the proteins were localized in the plastids, cytoplasm/cytosol. (C) Molecular function. The enriched categories were without GO associated and constituents of the ribosome.

**Fig 4:**
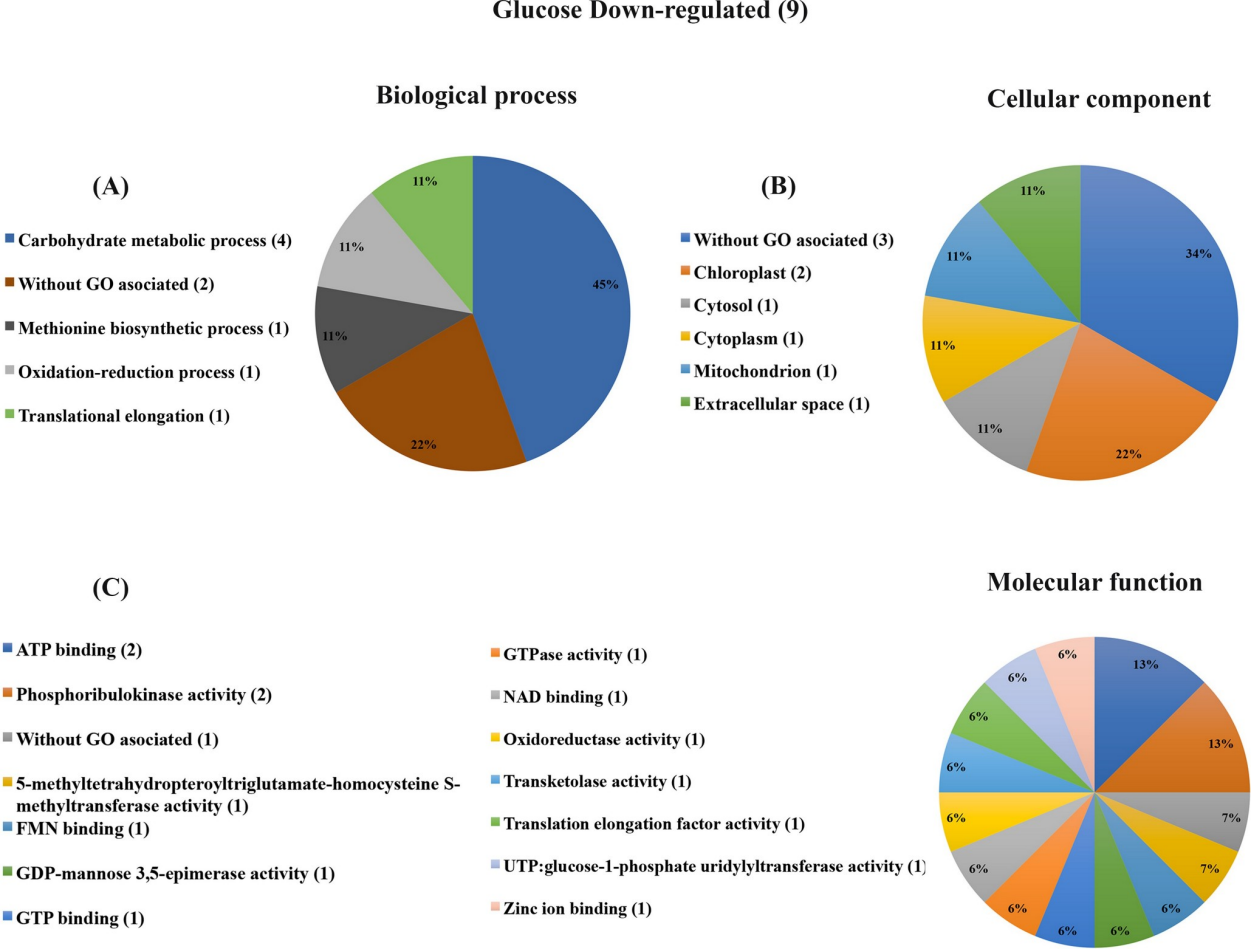
Graphical representation of gene ontology classification for proteins down-regulated by glucose treatment. (A) Biological process classification. Proteins involved carbohydrate metabolic process category were predominant. (B) Cellular component. The enriched category was without GO associated and chloroplast. (C) Molecular function. There was no evident enriched category.

Proteins more abundant in response to glucose were classified according to their biological process. The category with the highest number of proteins was translation (GO:0006412) with 8 proteins that include structural ribosomal proteins, a protein related to translation elongation (GO:0006414), as well as three nascent polypeptide-associated predicted proteins that bind to ribosomes (without GO associated: A9RHV4, A9SV00, A9U4U1) (Table 1). These 12 proteins related to the translation process represent 27% of the 44 proteins more abundant in high glucose compared to control treatment (Table 1; Figs 3A and 6). The second enriched category was photosynthesis, containing five proteins that include three proteins from the photosystem I reaction center and two Ribulose bisphosphate carboxylase small chain proteins. Other photosynthesis-related proteins correspond to the electron transport chain that includes two Plastocyanins, as well as one predicted protein classified in the Tetrapyrrole biosynthetic process. Taking together these eight photosynthesis-related proteins, represent 18% (Table 1; Figs 3A and 6). Other categories represented in this analysis were cellular response to oxidative stress (GO:0034599) which included one Peroxiredoxin and two Superoxide dismutases; oxidation-reduction process (GO:0055114) with one Monodehydroascorbate reductase III, and two predicted proteins (without GO associated: A9SVT2, A9TVV6) with glutathione S-transferase activity (according to UniProt and STRING databases), that result in six proteins that represent 13% of the glucose increased proteins. Protein refolding was another category represented by three proteins that include one Peptidyl-prolyl-cis-trans isomerase and two proteins that belong to the heat shock protein 70 family, as well as another heat shock protein belonging to the same family involved in the cellular response to heat category (according to STRING database), these four proteins represent 9% (Table 1; Figs 3A and 6). Additional categories such as ATP synthesis coupled proton transport and fatty acid biosynthetic process included two proteins each. The rest of the categories contain only one protein (Table 1 and Fig 3A). On the other hand, the most numerous category of the less-abundant proteins in response to glucose treatment was the carbohydrate metabolic process with four proteins (Table 1 and Fig 4A). It is worth noting that 20% of the 53 identified proteins in response to glucose have no biological process GO annotated in UniProt database.

According to the cellular component classification, *P*. *patens* proteins that increased after glucose feeding included 13 proteins associated with plastids, representing 21% (five in the chloroplast thylakoid membrane, three in the chloroplast, three in photosystem I reaction center and two in plastids); 11 cytoplasm and cytosol localized proteins (seven and four proteins respectively) corresponding to 18.6%, and 11 constituents of ribosome translation machinery proteins (three cytosolic small ribosomal subunits, three large ribosomal subunits, three nascent polypeptide associated complex and two cytosolic large ribosomal subunits), representing 18.3% of the proteins (Fig 3B). Other cellular component categories identified in this analysis with few proteins were found like mitochondrion, extracellular space, and membrane (Fig 3B). In the case of proteins with less abundance in the high glucose condition in comparison to the control, no clear enriched category was found possibly due to the reduced number of proteins (Fig 4B). It is important to stand out that 11 proteins more (eight) and less (three) abundant do not have a GO associated with a cellular component (Figs 3B and 4B). Regarding the molecular function classification, the enriched functions were related to ribosome/translation components representing 14.8% of the proteins with assigned GO (eight proteins, five corresponding to structural constituent of ribosome, two to large ribosomal subunit rRNA binding and one translation elongation factor activity) (Fig 3C). In the less abundant proteins, there was no evident enriched category (Fig 4C). Similarly to the biological process and cellular component classification, several proteins have no GO assigned to molecular function (nine and one more and less abundant, respectively). It is worth noting that in the biological process, cellular component, and molecular function classifications, the proteins related to translation are the most represented categories in response to glucose.

**Fig 5:**
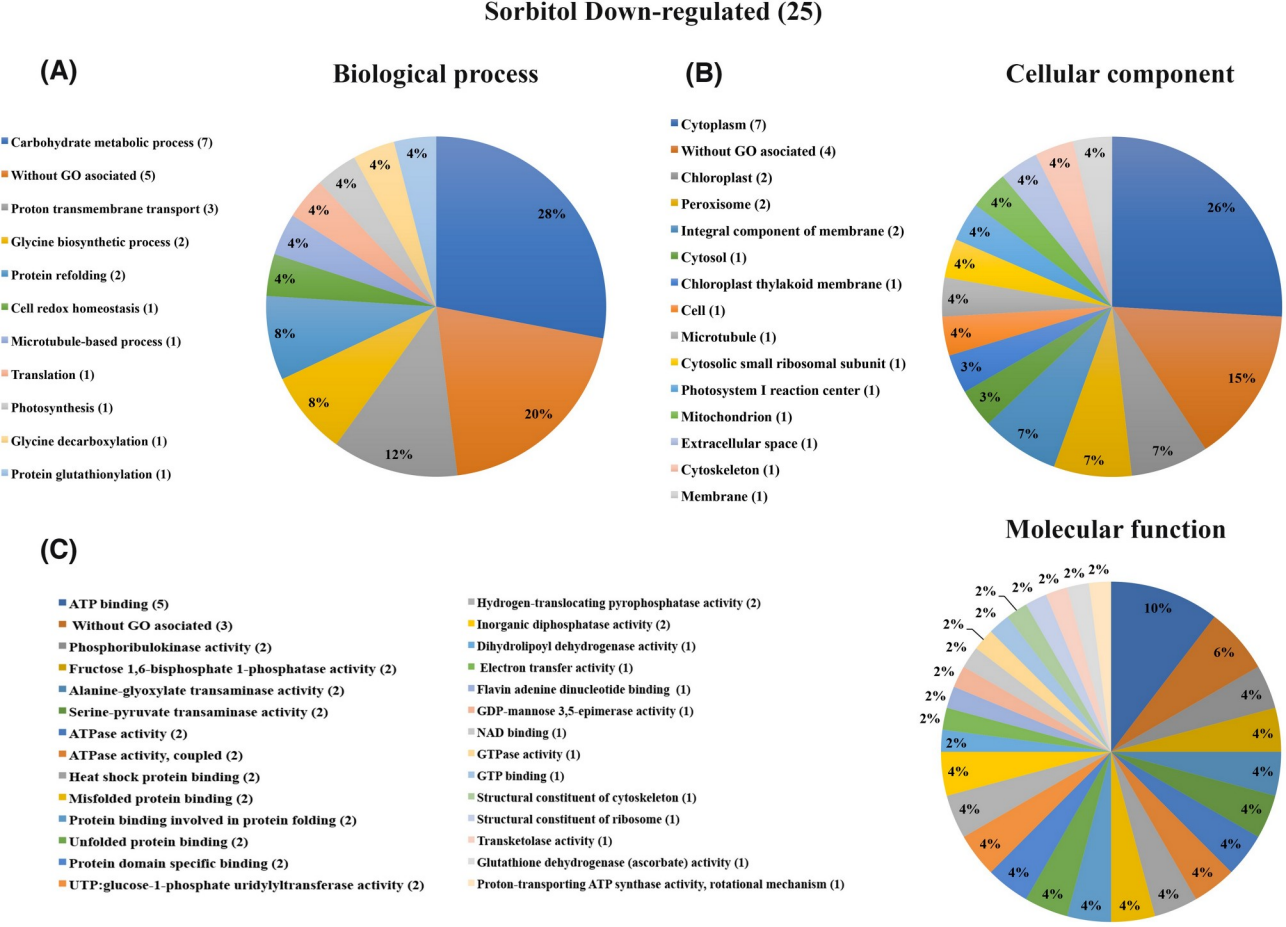
Graphical representation of gene ontology classification for proteins down-regulated by sorbitol treatment. (A) Biological processes. Proteins involved in carbohydrate metabolic process and proton trans-membrane transport were predominant. (B) Cellular component. The enriched localizations were cytoplasm/cytosol and proteins related to Plastids. (C) Molecular function. The enriched functions were related to ATP activities and proteins-folding functions.

Concerning the osmotic control, sorbitol-responsive proteins were classified according to biological processes and only one protein was more abundant, although it has no GO assigned (Table 2). In contrast, among the 25 decreased proteins, the enriched biological processes were carbohydrate metabolic process with seven proteins (representing the 28% of the total of 25 less abundant proteins); and proton transmembrane transport (with three proteins that represents the 12%) (Table 2 and Fig 5A). Interestingly, four of the proteins involved in carbohydrate metabolic process are common to the less abundant proteins in response to glucose, as well as two of the proteins without GO biological process associated (Tables 1 and 2), suggesting that the glucose response might be partially an osmotic effect, although the sorbitol treatment seems to have a stronger effect. Eight of the 25 less abundant proteins identified in the sorbitol treatment (which represented 30%) were localized in cytoplasm and cytosol (seven and one respectively), followed by four (representing 15%) chloroplastic proteins (two in the chloroplast, one in the chloroplast thylakoid membrane and one in photosystem I reaction center) (Table 2 and Fig 5B). Regarding the molecular function classification, the most numerous category was ATP related activities with 10 proteins (representing 21%), followed by six proteins (representing 12.5%) related to protein folding functions with two in Misfolded protein binding, two in Protein binding involved in protein folding and two in Unfolded protein binding) (Fig 5C). Other molecular function GO categories were found with one or two proteins (Fig 5C). Similarly to glucose-responsive proteins, an important fraction of proteins that respond to sorbitol does not have GO associated with biological processes, cellular components, or molecular functions (five, four, and three proteins respectively) (Fig 5).

**Fig 6:**
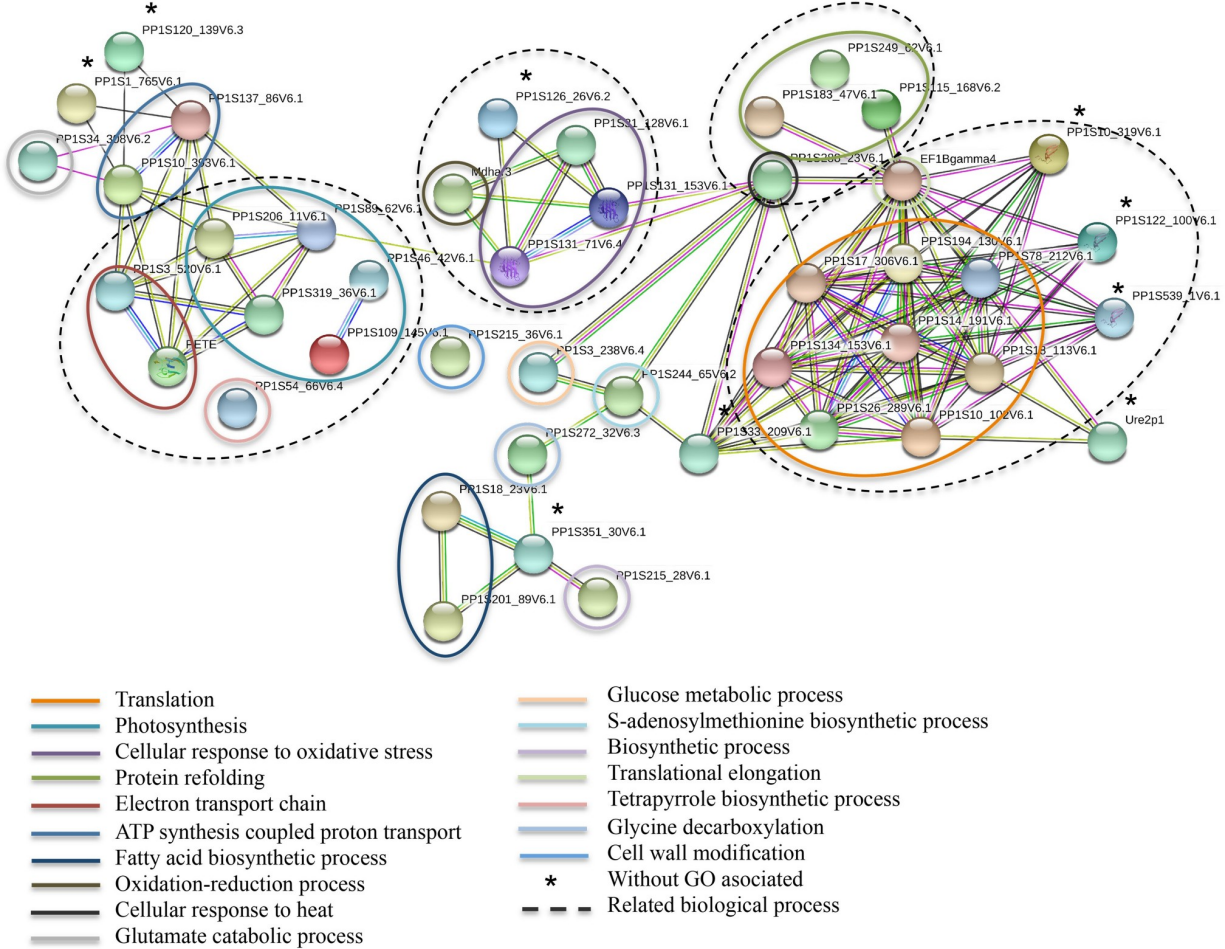
Functional protein association networks based on STRING. Analysis of *P*. *patens* proteins up-regulated in response to glucose. The lines connecting proteins represent: cyan, curated databases; magenta, experimentally determined; green, gene neighborhood; red, gene fusions; blue, gene co-occurrence; light green, textmining; black, co-expression; mauve, protein homology. Colored circles highlight biological processes.

Taken together, our results indicate that glucose induces specific changes in the proteome, including proteins mainly localized in plastids, cytoplasm/cytosol and ribosome-associated, highlighting the importance of these cellular components in response to glucose stimuli (Figs 3 and 4). Functional protein association networks (STRING), which integrate experimental, co-expression, and co-occurrence among other pieces of evidence, support these findings (Fig 6 and S4 Fig). In summary, our proteomic approach provided evidence that the *P*. *patens* glucose feeding experiments induced proteins mainly involved in translation, photosynthesis, cellular responses to oxidative stress, and protein refolding processes.

### High glucose levels did not impact the maximal rate of PSII

**Fig 7:**
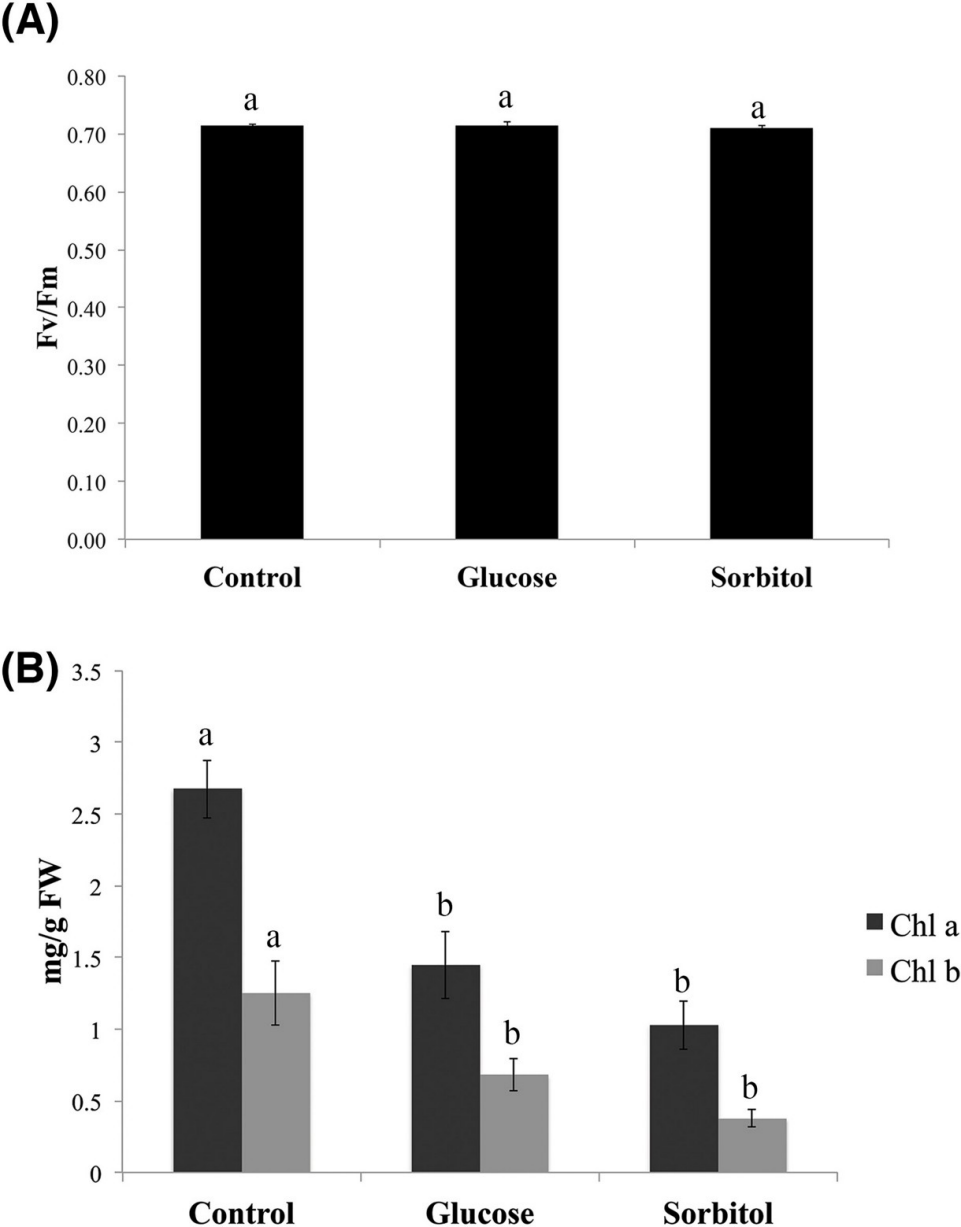
*P*. *patens* maximal rate of PSII and chlorophyll levels upon exposure to glucose and sorbitol. (A) Measurements of the chlorophyll fluorescence parameter (variable fluorescence [Fv]/maximal fluorescence [Fm]) were carried out at 24 h after the treatment of *P*. *patens* with glucose and sorbitol. The maximal rate of PSII was no affected by both treatments. (B) The absorption spectra of chlorophyll a and b were measured at 663 and 645 nm, respectively. Chlorophyll concentrations are expressed as mg chlorophyll per g dry weight (mg/g DW). Graphical representation of three independent biological samples means ± SE. Different letters indicate statistically significant differences (*P* ≤0.05) using a post hoc Tukey test (SAS university edition).

As some glucose-induced photosynthesis-related proteins were found (Table 1), we wondered if in *P*. *patens* the maximal rate of PSII was affected by glucose feeding treatment. Measurement of the Fv/Fm parameter showed that neither glucose nor sorbitol affected the maximal rate of PSII during the first 24 h of treatment (Fig 7A). However, a decrease in chlorophyll content was observed after glucose and sorbitol treatment (Fig 7B).

## Discussion

Although various sugars have emerged as important regulators during all stages of vascular plant development, glucose is the most prominent and evolutionarily conserved [4, 9, 10, 13, 25, 50–55]. Sugar concentrations ranging from 100 to 333 mM have been successfully used for *Arabidopsis* mutant screens in addition to gene expression assays [4, 18, 23]. In *P*. *patens*, 50–150 mM glucose also has been shown to have an effect [34, 35] (unpublished own data). In this study, we assessed the effect of high glucose (300 mM) in the non-vascular *P*. *patens* plant in an effort to understand its nutritional and/or metabolic role and distinguish these roles from glucose-induced osmotic effects. Omics strategies are powerful tools that provide integral information regarding global molecular changes in response to both internal and environmental influences. *P*. *patens* was then subject to exogenous high glucose concentration followed by metabolomic and proteomic analyses to determine global changes in the metabolism and protein population.

Several methodologies have been used to analyze metabolic phenotyping such as mass spectrometry (MS), liquid chromatography (LC), gas chromatography (GS), electron impact (EI), or the combination of these techniques (GC-MS or GC-EI-MS). However, the time it takes to analyze a single sample and the derivatization that some molecules need represent a great disadvantage. In this sense, the use of the direct-injection electrospray ionization-mass spectrometry (DIESI-MS) analytical technique favors an efficient ionization of hydrophilic metabolites, avoids compounds volatility, overcomes the need of chromatographic separation and the obtaining of multiple peaks resulting in data redundancy due to chemical derivatization, as well as overwhelms convoluted data workflow and statistical handling; resulting in a reliable, sensitive, and quantitative detection [39]. On the other hand, the first step in performing proteomics is to determine the number of proteins to be measured. In some cases, a defined set of proteins may be of interest to examine, so a targeted approach should be used [56]. In other cases, an untargeted approach, also known as “shotgun” approach, may be taken to measure as many proteins as possible and compared between samples without bias [57]. Most of the untargeted proteomic studies for the identification of proteins in *P*. *patens* make use of two-dimensional electrophoresis (2-DE) separation, followed by isolation of the differential spots and a Mass-spectrometry analysis [58–62]. Although the 2-DE analysis in combination with MS, is an untargeted approach, the main limitations of 2-DE separation are that many protein spots are not stainable with coomassie or silver, as well as identification of high molecular mass proteins results difficult, making the 2-DE approach less suitable for large-scale comparative protein expression studies [58, 63]. Recently, new methodologies with an improved sensitivity have emerged to detect proteins without the use of 2-DE. Among them, the label-free LC-MS analytical platform has increased its popularity in recent years due to the elimination of time-consuming stages for labeling proteins and the high number of proteins that can be detected [41, 63–66]. Thus, a label-free LC_MS Proteomics Approach was used in this study.

### Feeding of glucose and sorbitol caused specific metabolic responses in *P*. *patens*

The metabolomic fingerprinting showed that glucose feeding produced a global impact on *P*. *patens* metabolism (Fig 1 and S1–S3 Figs). The response to glucose feeding was distinct from that seen with the control and sorbitol treatments. Not all metabolites increased as a result of high glucose feeding; in fact, some decreased, which might indicate the activation of several primary metabolic enzymes. Several metabolites increased specifically in response to sorbitol feeding (Fig 1 and S1–S3 Figs). The ability of the moss *P*. *patens* to partially utilize sorbitol as a carbon source might provide one explanation for this finding. The route of carbon entry via sorbitol dehydrogenase (SDH) and a uridine phosphate-dependent fructokinase (FK) cannot be ruled out completely in this moss. In plant species of the *Rosaceae* family (such as apple), sorbitol metabolism represents a major carbon flow pathway [67]. However, fructose levels did not increase after sorbitol feeding (Fig 2), which can indicate that in *P*. *patens*, the total entry of carbon into the fructose pool via SDH was relatively low [68]. It is indisputable, though, that the entry of glucose proceeds via a plasma membrane-associated hexose carrier (HC), HK, and Phosphoglucoisomerase (PGI), which consumes cytosolic ATP and does not generate nicotinamide adenine dinucleotide (NADH). The entry route of glucose is different from the sorbitol entry route via SDH that generates redox equivalents in the form of NADH. FK may also consume UTP instead of ATP [69]. It is possible that carbon signaling is not sensing glucose levels per se, but rather it may measure activities such as carbon fluxes through the HC-HK-PGI pathway, which consumes large amounts of ATP [70].

### Glucose feeding impacts carbohydrate metabolism in *P*. *patens*

Since glucose is the primary source of energy, any change or imbalance in glucose availability can affect different cellular functions [10, 52, 71]. High glucose feeding caused specific changes in the metabolomic fingerprints of the moss *P*. *patens* (as shown in Fig 1 and S1–S3 Figs), and also evidenced by carbohydrate content alterations such as the increase in hexose levels, and decreasing sucrose and starch (Fig 2). Compared to control and sorbitol conditions, glucose feeding produced a decrease in sucrose content and significantly altered the hexose to starch ratio (Fig 2). It appears that starch was being remobilized by activating starch degradation or via starch synthesis inhibition through redox-regulated key enzymes such as α-glucan water dikinase (GWD1), stromal β-amylase (BAM1), α-amylase 3 (AMY3), Isoamylase 1 (ISA1), Isoamylase 2 (ISA2, DBE1), limit-dextrinase (LDA), and ADP-glucose phosphorylase (AGPase) [72]. Indeed, changes in carbohydrate metabolism are supported by the identification of differential proteins in the glucose treatment, such as glyceraldehyde-3-phosphate dehydrogenase (GAPDH), phosphoribulokinases (PRK), UTP-glucose-1-phosphate uridylyltransferase and a predicted protein (A9SF03) with transketolase activity (of the pentose phosphate pathway, according to UniProt database) (Table 1). Besides, the identification of a dihydrolipoamide acetyltransferase component of pyruvate dehydrogenase complex (dihydrolipoyllysine-residue acetyltransferase activity), suggests that acetyl-CoA production occurs during high glucose exposure. Since acetyl-CoA is committed to de novo fatty acid biosynthesis, this is likely a mechanism for relieving the carbonated molecule excess within the system [15, 73]. Also, we found other lipid metabolism proteins such as biotin carboxylase and one predicted protein (A9TC15) that belongs to beta-ketoacyl-ACP synthase family (3-oxoacyl-[acyl-carrier-protein] synthase activity) (Table 1), which supports this hypothesis.

### Glucose stimulates the accumulation of translation machinery proteins

An unexpected finding in the proteomic analysis was that translation category in the biological process classification was the most enriched, with 8 structural small and large ribosomal protein subunits besides a protein related to translation elongation and three nascent polypeptide-associated predicted proteins that bind to ribosomes, thus giving a total of 12 proteins which represent 27% of the proteins increased in response to glucose (Table 1). Multiple interaction evidences were revealed among this group of proteins by STRING functional association networks (Fig 6). Interestingly, Price et al. (2004) [18] suggested that gene induction by glucose feeding requires de novo protein synthesis in *Arabidopsis*, which is in agreement with our findings in *P*. *patens*. Several pieces of evidence further support the role of sugars in the regulation of transcript stability and processing, selective mRNA translation, ribosome biogenesis, mRNA polysome loading, translational activity, protein stability/degradation, and modulation of enzymatic activities, in plant growth and development control [7, 8, 13, 74]. This indicates that glucose exerts its effects beyond transcriptional gene regulation and includes multiple post-transcriptional regulatory mechanisms like stimulation of protein synthesis and/or stability. It has been shown that glucose-TOR signaling regulates transcription of genes related to central carbon and energy metabolism (glycolysis, TCA cycle, mitochondrial energy functions) and importantly ribosomal proteins, as well as protein synthesis machinery [6–8]. The role of sugar feeding and higher energetic status on translation regulation processes has been widely studied mainly in Arabidopsis, evidencing complex links between global and gene-specific translational control and chromatin activity. Particularly, upon sucrose concentration increase, certain ribosomal protein mRNAs are enriched in polysomes, and differential phosphorylation of ribosomal proteins occurs under high energetic conditions (reviewed in [75]. Also, the expression level of several ribosome biogenesis related genes is increased upon sugar feeding in Arabidopsis [76] yeast and mammalian cells [77, 78]. In addition, transcription of multiple ribosome proteins as well as rRNA is accelerated in plant cells thought the TOR-S6K signaling pathway [79, 80]. Outstandingly, Maekawa et al., (2018) [74] demonstrated important links among ribosome biogenesis, nucleolar stress, and sugar responses in plants through the study of the glucose-inducible nucleolus-localized APUM24 protein, which was shown to be involved in the control of Arabidopsis development by regulating ribosome biogenesis [81]. Despite the importance of these processes and their physiological impact, the molecular mechanisms are still unclear and pending for future research, particularly in non-vascular plants.

### Proteins involved in the cellular response to oxidative stress are increased upon glucose feeding

Although glycolysis is the most important metabolic pathway for producing cellular energy sources (such as NADPH and ATP in heterotrophs), it has been reported that sugar degradation by this pathway produces reactive carbonyls (RCs) as by-products [82–86]. Also, sugar auto-oxidation produces superoxide radicals (O_2_^-^) that are rapidly converted into hydrogen peroxide (H_2_O_2_) and oxygen (O_2_) by superoxide dismutase (SOD) [83]. The Fenton reaction catalyzes the conversion of these products into hydroxyl radicals (OH), which are the most potent form of ROS [84]. Although ROS are produced during normal cell metabolism during the life cycle in all organisms, an increase in ROS levels is also due to plant hormones, environmental stress, pathogens, and altered soluble sugar levels [3, 87]. Besides stimulating the anti-oxidant system soluble sugars by themselves might also act as ROS scavengers [88]. Consequently, ROS may also induce anti-oxidant systems, such as scavenging and other protective mechanisms [89]. Oxidative stress in plants is counteracted by the use of a range of ROS scavengers such as SOD, glutathione transferases (GST, molecular function predicted for A9SVT2 and A9TVV6 by UniProt and STRING databases) and peroxiredoxins (PRX) all identified as differentially accumulated in our proteomic analysis (Table 1). In *P*. *patens*, SOD could constitute one of the anti-oxidative defense strategies in conjunction with PRX and glutathione peroxidases (GPX) to reduce H_2_O_2_ levels and prevent cellular damage [90–95]. Ascorbate peroxidase (APX) activity, as affected by ascorbate (specific electron donor), results in the accumulation of monodehydro-ascorbate, which is reduced by monodehydro-ascorbate reductase (MDHAR) and is important for maintaining proper cellular ascorbate levels via NADPH as an electron donor [96–100]. In *Arabidopsis*, the expression of genes coding for MDHAR is induced by sugars [23]. Interestingly, our data indicate that this protein is also more abundant in response to glucose feeding in *P*. *patens* (Table 1). Thus, the ascorbate-glutathione cycle seems to be activated to prevent the potential ROS-derived cellular damage in response to high glucose levels. In *P*. *patens* and vascular plants, components of the anti-oxidative system have been identified in response to high salinity, desiccation, and ABA [59, 60, 62, 101–104]. Hence, growing evidence strongly suggests that the generation of ROS is one of the most common plant responses to different abiotic stresses. In conclusion, high glucose conditions apparently induce oxidative stress responses in *P*. *patens*, a model that possesses diverse strategies to counteract this condition.

### The regulation of photosynthesis in response to feeding glucose

In addition to photosynthesis, chloroplasts also host other metabolic reactions such as amino acid biosynthesis, hormones, vitamins, lipids, and secondary metabolites [105]. Thus, any disturbance in chloroplasts is communicated to the nucleus through retrograde signals to adjust all cellular activities [106]. In *Arabidopsis*, it is well-known that glucose accumulation/feeding results in the down-regulation of photosynthesis-associated genes, causing a decline in photosynthetic capacity [4, 6, 18, 24, 51, 52, 71, 107, 108]. Surprisingly, *P*. *patens* maintains normal levels of the maximal rate of PSII after 24 h of high glucose treatment, which coincides with the high number of photosynthesis-related proteins, that represent 18% of the identified more abundant proteins in response to glucose (Table 1 and Figs 3A and 6). Interestingly, two-electron transport chain chloroplastic proteins were highly induced in response to glucose (Table 1). This indicates that under high glucose levels *P*. *patens* chloroplasts are not affected on photosynthetic activity. It is worth noting that a high number of plastid proteins (13) were identified as differentially accumulated (Table 1 and Fig 3B). Photosynthetic pigments such as chlorophyll a and b decreased during glucose and sorbitol exposure (Fig 7B), suggesting that in *P*. *patens* these parameters are more sensitive to high glucose than the maximal rate of PSII, also indicating that the moss is not under optimal operating conditions. In contrast to vascular plants [109–112], *P*. *patens* seems to be less sensitive to osmotic- and glucose-induced photosynthetic inhibition. The biological significance of these differences in photosynthetic activities between vascular and non-vascular plants in response to glucose deserves deeper research. Altogether, our proteomic profile resulting from high glucose feeding suggests that this sugar activates the antioxidant system to protect cells from ROS-derived damage, especially for the photosynthetic machinery.

### Protein refolding has a relevant role during glucose response in *P*. *patens*

Other important proteins identified during sugar feeding experiments in *P*. *patens* were related to protein refolding, with four proteins that represent 9% of the more abundant proteins with the highest fold change: one Peptidyl-prolyl-cis-trans isomerase (14.07 fold change) and proteins that belong to the heat shock protein 70 family (A9ST56 with 11.75 and A9T8E8 with 8.86 fold changes), as well as another heat shock protein belonging to the same family (13.66 fold change) involved in the cellular response to heat category (Table 1; Figs 3A and 7). All these proteins bind to unfolded or misfolded proteins acting as chaperones that stabilize non-native polypeptides to suppress protein aggregation [113–115]. Consistent with their putative role in glucose-derived stress responses, HSP70 has been shown to be the major chaperone under abiotic stress responses, including those induced by high salinity, desiccation, cold, and high glucose concentrations [18, 22, 59, 60, 62, 116–118]. All of these abiotic stresses are tightly coupled to ABA and sugar-accumulating conditions. In summary, the *P*. *patens* proteomic response to high glucose levels seems to be closely related to stress responses.

This study represents just a first approach (proteomic and metabolomic) and the beginning of the study of sugar responses in non-vascular plants, definitely further studies are required to validate predicted proteins because most of the Physcomitrella proteins have not been characterized. Although transcript accumulation has been used in some reports to validate proteomic results, it is clear that the probability to correlate protein and transcript levels is very low due to very well-known multiple and rapid post-transcriptional and post-translational regulation levels [119–122].

## Conclusions

In this study, we explored the metabolomic and proteomic responses of the non-vascular plant, *P*. *patens*, to high glucose levels. We found that glucose feeding causes specific changes in moss metabolomic fingerprint, carbohydrate contents, and protein accumulation, which differed from osmotically induced responses. Our most significant discovery at the proteome level is that high glucose induced ribosomal proteins related to the translation process. It is worth noting that in the biological process, cellular component, and molecular function classifications, the categories including proteins related to translation are the most represented in response to glucose. Consistently, it is known that in plants such as *Arabidopsis* that growth and development responses to sugars are dependent on de novo protein synthesis and mRNA translation; however, this has not been previously evidenced in non-vascular plants. Moreover, the fact that glucose-induced proteins related to oxidative stress accumulate in *P*. *patens* under high glucose treatment, suggests that this plant possess an efficient ROS scavenging system. This idea is supported by the results showing that the glucose treatment did not alter the maximal rate of PSII and the electron transport chain. In summary, even though *A*. *thaliana* and *P*. *patens* represent two evolutionary distant plant lineages, the fact that glucose feeding affects the translational level of regulation in both model plants supports that a partially conserved response to glucose might exist between vascular and non-vascular plants. On the other hand differential responses may well be explained by the distant phylogenetic relationship between both plant species, such mechanisms are pending for future research, particularly in mosses.

## Supporting information

S1 Fig Ions obtained in *P*. *patens* protonemata exposed to glucose and sorbitol treatments. Protonemata were exposed to 300 mM of either glucose or sorbitol for 24 h. The ions were grouped into categories with their respective mass charge ratio (mz value) using statistical data mining with *P*<0.05. Results shown correspond to three independent biological samples.
(PDF)

S2 Fig Heatmap profile of 58 significant negative ions in response to glucose and sorbitol. *P*. *patens* protonemata were exposed to 300 mM of either glucose or sorbitol for 24 h. An optimized hierarchical clustering based on correlation R was applied to 58 significant negative ions. The metabolomic fingerprint is represented as a grayscale barcode that depicted the relative intensity (ion abundance), black indicates high and white indicates low. Ion similarity is revealed by the left dendrogram. Results shown correspond to three independent biological samples.
(PDF)

S3 Fig Heatmap profile of 50 top significant positive ions in response to the different treatments. *P*. *patens* protonemata were exposed to 300 mM of either glucose or sorbitol for 24 h. An optimized hierarchical clustering based on correlation R was applied to 50 top intensity significant positive ions. The metabolomic fingerprint is represented as a grayscale barcode that depicted the relative intensity (ion abundance), black indicates high and white indicates low. Ion similarity is revealed by the left dendrogram. Results shown correspond to three independent biological samples.
(PDF)

S4 Fig Functional protein association networks based on STRING. (A) Analysis of proteins relatively less abundant in response to glucose. (B) Proteins relatively less abundant in response to sorbitol (Q8H932 protein was not shown by the STRING database analysis). The lines connecting proteins are; Cyan, curated databases; magenta, experimentally determined; green, gene neighbourhood; red, gene fusions; blue, gene co-occurrence; light green, textmining; black, co-expression; mauve, protein homology. Colored circles highlight biological processes.
(PDF)

S1 Table Ions with a given common behaviour compared to control conditions. Values indicate the mass-charge ratio of the ion (mz value).
(XLSX)

S2 Table Proteins identified in response to glucose, sorbitol, and control treatments. (XLSX)

## References

[1] Yuan HX, Xiong Y, Guan KL. Nutrient Sensing, Metabolism, and Cell Growth Control. Mol Cell. 2013;49(3):379–87. 10.1016/j.molcel.2013.01.019 23395268 PMC3587157

[2] Efeyan A, Comb WC, Sabatini DM. Nutrient-sensing mechanisms and pathways. Nature. 2015;517(7534):302–10. 10.1038/nature14190 25592535 PMC4313349

[3] Couée I, Sulmon C, Gouesbet G, El Amrani A. Involvement of soluble sugars in reactive oxygen species balance and responses to oxidative stress in plants. J Exp Bot. 2006;57(3):449–59. 10.1093/jxb/erj027 16397003

[4] Ramon M, Rolland F, Sheen J. Sugar Sensing and Signaling. Arab B. 2008;6:e0117 10.1199/tab.0117 22303242 PMC3243355

[5] Rosa M, Prado C, Podazza G, Interdonato R, González JA, Hilal M, et al Sugars and Stress. 2009;4(5):388–93. 10.4161/psb.4.5.8294 PMC2676748 19816104

[6] Sheen J. Master regulators in plant glucose signaling networks. J Plant Biol. 2014;57(2):67–79. 10.1007/s12374-014-0902-7 25530701 PMC4270195

[7] Sakr S, Wang M, Dédaldéchamp F, Perez-Garcia MD, Ogé L, Hamama L, et al The sugar-signaling hub: Overview of regulators and interaction with the hormonal and metabolic network. Int J Mol Sci. 2018;19(9). 10.3390/ijms19092506 30149541 PMC6165531

[8] Lastdrager J, Hanson J, Smeekens S. Sugar signals and the control of plant growth and development. J Exp Bot. 2014;65(3):799–807. 10.1093/jxb/ert474 24453229

[9] Smeekens S. Sugar-induced signal transduction in plants. Annu Rev Plant Physiol Plant Mol Biol. 2000 6 1;51(1):49–81. 10.1146/annurev.arplant.51.1.49 15012186

[10] Rolland F, Moore B, Sheen J. Sugar Sensing and Signaling in Plants. Plant Cell. 2002;185–205. 10.1105/tpc.010455 12045277 PMC151255

[11] Rook F, Bevan MW. Genetic approaches to understanding sugar-response pathways. J Exp Bot. 2003;54(382):495–501. 10.1093/jxb/erg054 12508060

[12] Gibson SI. Control of plant development and gene expression by sugar signaling. Curr Opin Plant Biol. 2005;8(1):93–102. 10.1016/j.pbi.2004.11.003 15653406

[13] Rolland F, Baena-Gonzalez E, Sheen J. Sugar sensing and signaling in plants: Conserved and Novel Mechanisms. Annu Rev Plant Biol. 2006;57(1):675–709. 10.1146/annurev.arplant.57.032905.105441 16669778

[14] León P, Sheen J. Sugar and hormone connections. Trends Plant Sci. 2003;8(3):110–6. 10.1016/S1360-1385(03)00011-6 12663220

[15] Casteel J, Miernyk JA, Thelen JJ. Mapping the lipoylation site of *Arabidopsis thaliana* plastidial dihydrolipoamide S-acetyltransferase using mass spectrometry and site-directed mutagenesis. Plant Physiol Biochem. 2011;49(11):1355–61. 10.1016/j.plaphy.2011.07.001 21798751

[16] Zhou L, Jang J, Jones TL, Sheen J. Glucose and ethylene signal transduction crosstalk revealed by an Arabidopsis glucose-insensitive mutant. Proc Natl Acad Sci. 1998;95(17):10294 LP–10299. 10.1073/pnas.95.17.10294 9707641 PMC21502

[17] Xiao W, Sheen J, Jang JC. The role of hexokinase in plant sugar signal transduction and growth and development. Plant Mol Biol. 2000;44(4):451–61. 10.1023/a:1026501430422 11197321

[18] Price J, Laxmi A, St. Martin SK, Jang JC. Global transcription profiling reveals multiple sugar signal transduction mechanisms in Arabidopsis. Plant Cell. 2004;16(8):2128–50. 10.1105/tpc.104.022616 15273295 PMC519203

[19] Cho YH, Yoo SD, Sheen J. Regulatory Functions of Nuclear Hexokinase1 Complex in Glucose Signaling. Cell. 2006;127(3):579–89. 10.1016/j.cell.2006.09.028 17081979

[20] Deprost D, Yao L, Sormani R, Moreau M, Leterreux G, Bedu M, et al The Arabidopsis TOR kinase links plant growth, yield, stress resistance and mRNA translation. EMBO Rep. 2007;8(9):864–70. 10.1038/sj.embor.7401043 17721444 PMC1973950

[21] Robaglia C, Thomas M, Meyer C. Sensing nutrient and energy status by SnRK1 and TOR kinases. Curr Opin Plant Biol. 2012;15(3):301–7. 10.1016/j.pbi.2012.01.012 22305521

[22] Thum KE, Shin MJ, Palenchar PM, Kouranov A, Coruzzi GM. Genome-wide investigation of light and carbon signaling interactions in Arabidopsis. Genome Biol. 2004;5(2):1–20. 10.1186/gb-2004-5-2-r10 PMC395748 14759260

[23] Han L, Li JL, Jin M, Su YH. Transcriptome analysis of Arabidopsis seedlings responses to high concentrations of glucose. Genet Mol Res. 2015;14(2):4784–801. 10.4238/2015.May.11.11 25966253

[24] Sami F, Yusuf M, Faizan M, Faraz A, Hayat S. Role of sugars under abiotic stress. Plant Physiol Biochem. 2016;109:54–61. 10.1016/j.plaphy.2016.09.005 27639065

[25] Smeekens S, Ma J, Hanson J, Rolland F. Sugar signals and molecular networks controlling plant growth. Curr Opin Plant Biol. 2010;13(3):273–8. 10.1016/j.pbi.2009.12.002 20056477

[26] Xu P, Kong Y, Li X, Li L. Identification of molecular processes needed for vascular formation through transcriptome analysis of different vascular systems. BMC Genomics. 2013;14(1):1 10.1186/1471-2164-14-217 23548001 PMC3620544

[27] Rensing SA, Lang D, Zimmer AD, Terry A, Salamov A, Shapiro H, et al The Physcomitrella genome reveals evolutionary insights into the conquest of land by plants. Science. 2008;319(5859):64–9. 10.1126/science.1150646 18079367

[28] Cho SH, von Schwartzenberg K, Quatrano R. The Role of Abscisic Acid in Stress Tolerance. Annu Plant Rev online. 2018;36:282–97. 10.1038/s41598-018-34862-1 30413758 PMC6226459

[29] Rensing SA, Goffinet B, Meyberg R, Wu SZ, Bezanilla M. The moss *Physcomitrium* (*Physcomitrella*) *patens*: A model organism for non-seed plants. Plant Cell. 2020;32(5):1361–76. 10.1105/tpc.19.00828 32152187 PMC7203925

[30] Schaefer DG, Zrÿd JP. The moss *Physcomitrella patens*, now and then. Plant Physiol. 2001;127(4):1430–8. 11743086 PMC1540175

[31] Benito B, Rodríguez-Navarro A. Molecular cloning and characterization of a sodium-pump ATPase of the moss *Physcomitrella patens*. Plant J. 2003;36(3):382–9. 10.1046/j.1365-313x.2003.01883.x 14617094

[32] Frank W, Ratnadewi D, Reski R. *Physcomitrella patens* is highly tolerant against drought, salt and osmotic stress. Planta. 2005;220(3):384–94. 10.1007/s00425-004-1351-1 15322883

[33] Saavedra L, Svensson J, Carballo V, Izmendi D, Welin B, Vidal S. A dehydrin gene in *Physcomitrella patens* is required for salt and osmotic stress tolerance. Plant J. 2006;45(2):237–49. 10.1111/j.1365-313X.2005.02603.x 16367967

[34] Olsson T, Thelander M, Ronne H. A Novel Type of Chloroplast Stromal Hexokinase Is the Major Glucose-phosphorylating Enzyme in the Moss *Physcomitrella patens*. J Biol Chem. 2003;278(45):44439–47. 10.1074/jbc.M306265200 12941966

[35] Thelander M, Olsson T, Ronne H. Effect of the energy supply on filamentous growth and development in *Physcomitrella patens*. J Exp Bot. 2005;56(412):653–62. 10.1093/jxb/eri040 15611148

[36] Azzabi G, Pinnola A, Betterle N, Bassi R, Alboresi A. Enhancement of non-photochemical quenching in the bryophyte *Physcomitrella patens* during acclimation to salt and osmotic stress. Plant Cell Physiol. 2012;53(10):1815–25. 10.1093/pcp/pcs124 22952250

[37] Agarwal T, Upadhyaya G, Halder T, Mukherjee A, Majumder AL, Ray S. Different dehydrins perform separate functions in *Physcomitrella patens*. Planta. 2017;245(1):101–18. 10.1007/s00425-016-2596-1 27638172

[38] García-Flores M, Juárez-Colunga S, Montero-Vargas JM, López-Arciniega JAI, Chagolla A, Tiessen A, et al Evaluating the physiological state of maize (*Zea mays* L.) plants by direct-injection electrospray mass spectrometry (DIESI-MS). Mol Biosyst. 2012;8(6):1658–60. 10.1039/c2mb25056j 22513980

[39] García-Flores M, Juárez-Colunga S, García-Casarrubias A, Trachsel S, Winkler R, Tiessen A. Metabolic profiling of plant extracts using direct-injection electrospray ionization mass spectrometry allows for high-throughput phenotypic characterization according to genetic and environmental effects. J Agric Food Chem. 2015;63(3):1042–52. 10.1021/jf504853w 25588121

[40] Vargas-Ortiz E, Espitia-Rangel E, Tiessen A, Délano-Frier JP. Grain Amaranths Are Defoliation Tolerant Crop Species Capable of Utilizing Stem and Root Carbohydrate Reserves to Sustain Vegetative and Reproductive Growth after Leaf Loss. PLoS One. 2013;8(7):1–13. 10.1371/journal.pone.0067879 23861825 PMC3701626

[41] Pando-Robles V, Oses-Prieto JA, Rodríguez-Gandarilla M, Meneses-Romero E, Burlingame AL, Batista CVF. Quantitative proteomic analysis of Huh-7 cells infected with Dengue virus by label-free LC-MS. J Proteomics. 2014;111:16–29. 10.1016/j.jprot.2014.06.029 25009145

[42] Nesvizhskii AI, Keller A, Kolker E, Aebersold R. A statistical model for identifying proteins by tandem mass spectrometry. Anal Chem. 2003;75(17):4646–58. 10.1021/ac0341261 14632076

[43] Ashburner M, Ball CA, Blake JA, Botstein D, Butler H, Cherry JM, et al The Gene Ontology Consortium. Nat Genet. 2000;25(1):25–9. 10.1038/75556 10802651 PMC3037419

[44] Wellburn AR. The Spectral Determination of Chlorophylls a and b, as well as Total Carotenoids, Using Various Solvents with Spectrophotometers of Different Resolution. J Plant Physiol. 1994;144(3):307–13. 10.1016/S0176-1617(11)81192-2

[45] Szklarczyk D, Franceschini A, Wyder S, Forslund K, Heller D, Huerta-Cepas J, et al STRING v10: Protein-protein interaction networks, integrated over the tree of life. Nucleic Acids Res. 2015;43(D1):D447–52. 10.1093/nar/gku1003 25352553 PMC4383874

[46] Cline MS, Smoot M, Cerami E, Kuchinsky A, Landys N, Workman C, et al Integration of biological networks and gene expression data using Cytoscape. Nat Protoc. 2007;2(10):2366–82. 10.1038/nprot.2007.324 17947979 PMC3685583

[47] Conesa A, Götz S, García-Gómez JM, Terol J, Talón M, Robles M. Blast2GO: A universal tool for annotation, visualization and analysis in functional genomics research. Bioinformatics. 2005;21(18):3674–6. 10.1093/bioinformatics/bti610 16081474

[48] Bindea G, Mlecnik B, Hackl H, Charoentong P, Tosolini M, Kirilovsky A, et al ClueGO: A Cytoscape plug-in to decipher functionally grouped gene ontology and pathway annotation networks. Bioinformatics. 2009;25(8):1091–3. 10.1093/bioinformatics/btp101 19237447 PMC2666812

[49] Tiessen A, Cubedo-Ruiz EA, Winkler R. Improved Representation of Biological Information by Using Correlation as Distance Function for Heatmap Cluster Analysis. Am J Plant Sci. 2017;08(03):502–16.

[50] Li L, Sheen J. Dynamic and diverse sugar signaling. Curr Opin Plant Biol. 2016 10;33:116–25. 10.1016/j.pbi.2016.06.018 27423125 PMC5050104

[51] Koch KE. Carbohydrate-modulated gene expression in plants. Annu Rev Plant Physiol Plant Mol Biol. 1996;47(1):509–40. 10.1146/annurev.arplant.47.1.509 15012299

[52] Koch K. Sucrose metabolism: Regulatory mechanisms and pivotal roles in sugar sensing and plant development. Curr Opin Plant Biol. 2004;7(3):235–46. 10.1016/j.pbi.2004.03.014 15134743

[53] Wobus U, Weber H. Sugars as signal molecules in plant seed development. Biol Chem. 1999;380(7–8):937–44. 10.1515/BC.1999.116 10494845

[54] Ohto M, Onai K, Furukawa Y, Aoki E, Araki T, Nakamura K. Effects of Sugars on Vegetative Development and Floral Transition. 2001;127:252–61. 10.1104/pp.127.1.252 PMC117981 11553753

[55] Eveland AL, Jackson DP. Sugars, signalling, and plant development. J Exp Bot. 2012;63(9):3367–77. 10.1093/jxb/err379 22140246

[56] Doerr A. Targeted proteomics. Nat Methods. 2011;8(1):43 10.1038/nmeth.f.329

[57] Zhang Y, Fonslow BR, Shan B, Baek MC, Yates JR. Protein analysis by shotgun/bottom-up proteomics. Chem Rev. 2013;113(4):2343–94. 10.1021/cr3003533 23438204 PMC3751594

[58] Sarnighausen E, Wurtz V, Heintz D, Van Dorsselaer A, Reski R. Mapping of the *Physcomitrella patens* proteome. Phytochemistry. 2004;65(11):1589–607. 10.1016/j.phytochem.2004.04.028 15276455

[59] Wang X, Yang P, Gao Q, Liu X, Kuang T, Shen S, et al Proteomic analysis of the response to high-salinity stress in *Physcomitrella patens*. Planta. 2008;228(1):167–77. 10.1007/s00425-008-0727-z 18351383

[60] Wang XQ, Yang PF, Liu Z, Liu WZ, Hu Y, Chen H, et al Exploring the mechanism of *Physcomitrella patens* desiccation tolerance through a proteomic strategy. Plant Physiol. 2009;149(4):1739–50. 10.1104/pp.108.131714 19211702 PMC2663739

[61] Polyakov NB, Slizhikova DK, Izmalkova MY, Cherepanova NI, Kazakov VS, Rogova MA, et al Proteome analysis of chloroplasts from the moss *Physcomitrella patens* (Hedw.) B.S.G. Biochem. 2010;75(12):1470–83. 10.1134/s0006297910120084 21314618

[62] Wang X, Kuang T, He Y. Conservation between higher plants and the moss *Physcomitrella patens* in response to the phytohormone abscisic acid: A proteomics analysis. BMC Plant Biol. 2010;10(192). 10.1186/1471-2229-10-192 20799958 PMC2956542

[63] Wang G, Wu WW, Zeng W, Chou CL, Shen RF. Label-free protein quantification using LC-coupled ion trap or FT mass spectrometry: Reproducibility, linearity, and application with complex proteomes. J Proteome Res. 2006;5(5):1214–23. 10.1021/pr050406g 16674111

[64] Levin Y, Bahn S. Quantification of proteins by Label-Free LC-MS/MS In Cutillas P, Timms J (eds) LC-MS/MS in Proteomics. Methods in Molecular Biology (Methods and Protocols). Humana Press, Totowa, NJ 2010;658:217–31. 10.1007/978-1-60761-780-8_13 20839107

[65] Bantscheff M, Schirle M, Sweetman G, Rick J, Kuster B. Quantitative mass spectrometry in proteomics: A critical review. Anal Bioanal Chem. 2007;389(4):1017–31. 10.1007/s00216-007-1486-6 17668192

[66] Nahnsen S, Bielow C, Reinert K, Kohlbacher O. Tools for label-free peptide quantification. Mol Cell Proteomics. 2013;12(3):549–56. 10.1074/mcp.R112.025163 23250051 PMC3591650

[67] Berüter J, Studer Feusi ME. Comparison of Sorbitol Transport in Excised Tissue Discs and Cortex Tissue of Intact Apple Fruit. J Plant Physiol. 1995;146(1–2):95–102.

[68] Lo Bianco R, Rieger M. Partitioning of sorbitol and sucrose catabolism within peach fruit. J Am Soc Hortic Sci. 2002;127(1):115–21.

[69] Stein O, Granot D. Plant fructokinases: Evolutionary, developmental, and metabolic aspects in sink tissues. Front Plant Sci. 2018;9:1–12. 10.3389/fpls.2018.00001 29616058 PMC5864856

[70] Aguilera-Alvarado GP, Sánchez-Nieto S. Plant Hexokinases are Multifaceted Proteins. Plant Cell Physiol. 2017;58(7):1151–60. 10.1093/pcp/pcx062 28449056

[71] Pego J V., Kortstee AJ, Huijser C, Smeekens SCM. Photosynthesis, sugars and the regulation of gene expression. J Exp Bot. 2000;51(suppl_1):407–16. 10938849 10.1093/jexbot/51.suppl_1.407

[72] Streb S, Zeeman SC. Starch Metabolism in Arabidopsis. Arab B. 2012;10(10):e0160. 10.1199/tab.0160 PMC3527087 23393426

[73] Broz AK, Tovar-Méndez A, Mooney BP, Johnston ML, Miernyk JA, Randall DD. A novel regulatory mechanism based upon a dynamic core structure for the mitochondrial pyruvate dehydrogenase complex. Mitochondrion. 2014;19:144–53. 10.1016/j.mito.2014.05.003 24846799

[74] Maekawa S, Ishida T, Yanagisawa S. Reduced expression of APUM24, encoding a novel rRNA processing factor, induces sugar-dependent nucleolar stress and altered sugar responses in *Arabidopsis thaliana*. Plant Cell. 2018;30(1):209–27. 10.1105/tpc.17.00778 29242314 PMC5810573

[75] Merchante C, Stepanova AN, Alonso JM. Translation regulation in plants: an interesting past, an exciting present and a promising future. Plant J. 2017;90(4):628–53. 10.1111/tpj.13520 28244193

[76] Kojima H, Suzuki T, Kato T, Enomoto K, Sato S, Kato T, et al Sugar-inducible expression of the nucleolin-1 gene of Arabidopsis thaliana and its role in ribosome synthesis, growth and development. Plant J. 2007;49: 1053–1063. 10.1111/j.1365-313X.2006.03016.x 17286797

[77] Powers T, Walter P. Regulation of ribosome biogenesis by the rapamycin-sensitive TOR-signaling pathway in *Saccharomyces cerevisiae*. Mol Biol Cell. 1999; (4):987–1000. 10.1091/mbc.10.4.987 10198052 PMC25225

[78] Iadevaia V, Liu R, Proud CG. mTORC1 signaling controls multiple steps in ribosome biogenesis. Semin Cell Dev Biol. 2014;36:113–20. 10.1016/j.semcdb.2014.08.004 25148809

[79] Xiong Y, McCormack M, Li L, Hall Q, Xiang C, Sheen J. Glucose-TOR signalling reprograms the transcriptome and activates meristems. Nature. 2013;496(7444):181–6. 10.1038/nature12030 23542588 PMC4140196

[80] Xiong Y, Sheen J. Novel links in the plant TOR kinase signaling network. Curr Opin Plant Biol. 2015;28:83–91. 10.1016/j.pbi.2015.09.006 26476687 PMC4612364

[81] Maekawa S, Yanagisawa S. Nucleolar stress and sugar response in plants. Plant Signal Behav. 2018;13(3):e1442975 10.1080/15592324.2018.1442975 29465318 PMC5927710

[82] Phillips SA, Thornalley PJ. The formation of methylglyoxal from triose phosphates. Investigation using a specific assay for methylglyoxal. Eur J Biochem. 1993;105:101–5. 10.1111/j.1432-1033.1993.tb17638.x 8444148

[83] Medina-Navarro R, Durán-Reyes G, Díaz-Flores M, Rodríguez JK, Hicks JJ. Glucose autoxidation produce acrolein from lipid peroxidation in vitro. Clin Chim Acta. 2003;337(1–2):183–5. 10.1016/j.cccn.2003.07.005 14568199

[84] Stadtman ER, Levine RL. Free radical-mediated oxidation of free amino acids and amino acid residues in proteins. Amino Acids. 2003;25(3–4):207–18. 10.1007/s00726-003-0011-2 14661084

[85] Vistoli G, De Maddis D, Cipak A, Zarkovic N, Carini M, Aldini G. Advanced glycoxidation and lipoxidation end products (AGEs and ALEs): An overview of their mechanisms of formation. Free Radic Res. 2013;47(S1):3–27. 23767955 10.3109/10715762.2013.815348

[86] Takagi D, Inoue H, Odawara M, Shimakawa G, Miyake C. The calvin cycle inevitably produces sugar-derived reactive carbonyl methylglyoxal during photosynthesis: A potential cause of plant diabetes. Plant Cell Physiol. 2014;55(2):333–40. 10.1093/pcp/pcu007 24406631 PMC3913449

[87] Ramel F, Sulmon C, Bogard M, Couée I, Gouesbet G. Differential patterns of reactive oxygen species and antioxidative mechanisms during atrazine injury and sucrose-induced tolerance in *Arabidopsis thaliana* plantlets. BMC Plant Biol. 2009;9:1–18. 10.1186/1471-2229-9-1 19284649 PMC2661893

[88] Van Den Ende W, Valluru R. Sucrose, sucrosyl oligosaccharides, and oxidative stress: Scavenging and salvaging? J Exp Bot. 2009;60(1):9–18. 10.1093/jxb/ern297 19036839

[89] Gill SS, Singh LP, Tuteja N, Gill R. Generation and Scavenging of Reactive Oxygen Species in Plants under Stress. Improv Crop Resist to Abiotic Stress. 2012;1:49–70.

[90] Anjum NA, Gill SS, Gill R, Hasanuzzaman M, Duarte AC, Pereira E, et al Metal/metalloid stress tolerance in plants: role of ascorbate, its redox couple, and associated enzymes. Protoplasma. 2014;251(6):1265–83. 10.1007/s00709-014-0636-x 24682425

[91] Perkins A, Poole LB, Karplus PA. Tuning of peroxiredoxin catalysis for various physiological roles. Biochemistry. 2014;53(49):7693–705. 10.1021/bi5013222 25403613 PMC4270387

[92] Sytykiewicz H. Differential expression of superoxide dismutase genes in aphid-stressed maize (*Zea mays* L.) seedlings. PLoS One. 2014;9(4). 10.1371/journal.pone.0094847 24722734 PMC3983269

[93] Sofo A, Scopa A, Nuzzaci M, Vitti A. Ascorbate peroxidase and catalase activities and their genetic regulation in plants subjected to drought and salinity stresses. Int J Mol Sci. 2015;16(6):13561–78. 10.3390/ijms160613561 26075872 PMC4490509

[94] Perkins A, Nelson KJ, Parsonage D, Poole LB, Karplus PA. Peroxiredoxins: guardians against oxidative stress and modulators of peroxide signaling. Trends Biochem Sci. 2015;40(8):435–45. 10.1016/j.tibs.2015.05.001 26067716 PMC4509974

[95] Ozyigit II, Filiz E, Vatansever R, Kurtoglu KY, Koc I, Öztürk MX, et al Identification and comparative analysis of H2O2-scavenging enzymes (ascorbate peroxidase and glutathione peroxidase) in selected plants employing bioinformatics approaches. Front Plant Sci. 2016;7:1–23. 10.3389/fpls.2016.00001 27047498 PMC4802093

[96] Conklin PL, Saracco SA, Norris SR, Last RL. Identification of Ascorbic Acid-Deficient *Arabidopsis thaliana*; Mutants. Genetics. 2000;154(2):847–856. 10655235 10.1093/genetics/154.2.847 PMC1460938

[97] Teixeira FK, Menezes-Benavente L, Margis R, Margis-Pinheiro M. Analysis of the molecular evolutionary history of the ascorbate peroxidase gene family: Inferences from the rice genome. J Mol Evol. 2004;59(6):761–70. 10.1007/s00239-004-2666-z 15599508

[98] Sano S, Tao S, Endo Y, Inaba T, Hossain MA, Miyake C, et al Purification and cDNA cloning of chloroplastic monodehydroascorbate reductase from spinach. Biosci Biotechnol Biochem. 2005;69(4):762–72. 10.1271/bbb.69.762 15849415

[99] Gallie DR. L-Ascorbic Acid: A Multifunctional Molecule Supporting Plant Growth and Development. Scientifica (Cairo). 2013;2013:1–24. 10.1155/2013/795964 24278786 PMC3820358

[100] Kao CH. Role of L-Ascorbic Acid in Rice Plants. Crop, Environment & Bioinformatics. 2015;12:1–7

[101] Foyer CH, Noctor G. Stress-triggered redox signalling: What’s in pROSpect? Plant Cell Environ. 2016;39(5):951–64. 10.1111/pce.12621 26264148

[102] Sharma P, Jha AB, Dubey RS, Pessarakli M. Reactive Oxygen Species, Oxidative Damage, and Antioxidative Defense Mechanism in Plants under Stressful Conditions. J Bot. 2012;2012:1–26.

[103] Choudhury S, Panda P, Sahoo L, Panda SK. Reactive oxygen species signaling in plants under abiotic stress. Plant Signal Behav. 2013;8(4). 10.4161/psb.23681 23425848 PMC7030282

[104] Foyer CH, Noctor G. Redox homeostasis and antioxidant signaling: A metabolic interface between stress perception and physiological responses. Plant Cell. 2005;17(7):1866–75. 10.1105/tpc.105.033589 15987996 PMC1167537

[105] Leister D. Chloroplast research in the genomic age. Trends Genet. 2003;19:47–56. 10.1016/s0168-9525(02)00003-3 12493248

[106] Bobik K, Burch-Smith TM. Chloroplast signaling within, between and beyond cells. Front Plant Sci. 2015;6:1–26. 10.3389/fpls.2015.00001 26500659 PMC4593955

[107] Krapp A, Hofmann B, Schäfer C, Stitt M. Regulation of the expression of rbcS and other photosynthetic genes by carbohydrates: A mechanism for the “sink regulation” of photosynthesis? Plant J. 1993;3(6):817–28.

[108] Tholen D, Pons TL, Voesenek LACJ, Poorter H. Ethylene insensitivity results in down-regulation of Rubisco expression and photosynthetic capacity in tobacco. Plant Physiol. 2007;144(3):1305–15. 10.1104/pp.107.099762 17535822 PMC1914117

[109] Woo NS, Badger MR, Pogson BJ. A rapid, non-invasive procedure for quantitative assessment of drought survival using chlorophyll fluorescence. Plant Methods. 2008;4(1):1–14. 10.1186/1746-4811-4-27 19014425 PMC2628343

[110] Chaves MM, Flexas J, Pinheiro C. Photosynthesis under drought and salt stress: Regulation mechanisms from whole plant to cell. Ann Bot. 2009;103(4):551–60. 10.1093/aob/mcn125 18662937 PMC2707345

[111] Saibo NJM, Lourenço T, Oliveira MM. Transcription factors and regulation of photosynthetic and related metabolism under environmental stresses. Ann Bot. 2009;103(4):609–23. 10.1093/aob/mcn227 19010801 PMC2707349

[112] Gao HJ, Lü XP, Zhang L, Qiao Y, Zhao Q, Wang YP, et al Transcriptomic profiling and physiological analysis of haloxylon ammodendron in response to osmotic stress. Int J Mol Sci. 2018;19(1). 10.3390/ijms19010084 PMC5796034 29286291

[113] Bukau B, Horwich AL. The Hsp70 and Hsp60 chaperone machines. Cell. 1998;92(3):351–66. 10.1016/s0092-8674(00)80928-9 9476895

[114] Hartl FU, Hayer-Hartl M. Protein folding. Molecular chaperones in the cytosol: From nascent chain to folded protein. Science. 2002;295(5561):1852–8. 10.1126/science.1068408 11884745

[115] Hennessy F, Nicoll WS, Zimmermann R, Cheetham ME, Blatch GL. Not all J domains are created equal: Implications for the specificity of Hsp40-Hsp70 interactions. Protein Sci. 2005;14(7):1697–709. 10.1110/ps.051406805 15987899 PMC2253343

[116] Wang X, Yang P, Zhang X, Xu Y, Kuang T, Shen S et al Proteomic analysis of the cold stress response in the moss, *Physcomitrella patens*. Proteomics. 2009;9(19):4529–38. 10.1002/pmic.200900062 19670371

[117] Ndimba BK, Chivasa S, Simon WJ, Slabas AR. Identification of Arabidopsis salt and osmotic stress responsive proteins using two-dimensional difference gel electrophoresis and mass spectrometry. Proteomics. 2005;5(16):4185–96. 10.1002/pmic.200401282 16254930

[118] Cruz De Carvalho R, Bernardes Da Silva A, Soares R, Almeida AM, Coelho AV, Marques Da Silva J, et al Differential proteomics of dehydration and rehydration in bryophytes: Evidence towards a common desiccation tolerance mechanism. Plant, Cell Environ. 2014;37(7):1499–515. 10.1111/pce.12266 24393025

[119] Haider S, Pal R. Integrated Analysis of Transcriptomic and Proteomic Data. Curr Genomics. 2013;14(2):91–110. 10.2174/1389202911314020003 24082820 PMC3637682

[120] Stare T, Stare K, Weckwerth W, Wienkoop S, Gruden K. Comparison between proteome and transcriptome response in potato (*Solanum tuberosum* L.) leaves following potato virus Y (PVY) infection. Proteomes. 2017;5(3). 10.3390/proteomes5030014 28684682 PMC5620531

[121] Ghazalpour A, Bennett B, Petyuk VA, Orozco L, Hagopian R, Mungrue IN, et al Comparative analysis of proteome and transcriptome variation in mouse. PLoS Genet. 2011;7(6). 10.1371/journal.pgen.1001393 PMC3111477 21695224

[122] Bai Y, Wang S, Zhong H, Yang Q, Zhang F, Zhuang Z, et al Integrative analyses reveal transcriptome-proteome correlation in biological pathways and secondary metabolism clusters in A. flavus in response to temperature. Sci Rep. 2015;5:1–13. 10.1038/srep14582 PMC4586720 26416011

