# Supplementary material for: High levels of glucose alter Physcomitrella patens metabolism and trigger a differential proteomic response
Source: PLoS One. 2020 Dec 4;15(12):e0242919. doi: 10.1371/journal.pone.0242919 (PMC7717569; doi:10.1371/journal.pone.0242919)
Supplement: S1 Fig — Protonemata were exposed to 300 mM of either glucose or sorbitol for 24 h. The ions were grouped into categories with their respective mass charge ratio (mz value) using statistical data mining with P<0.05. Results shown correspond to three independent biological samples. (PDF) [file pone.0242919.s001.pdf]

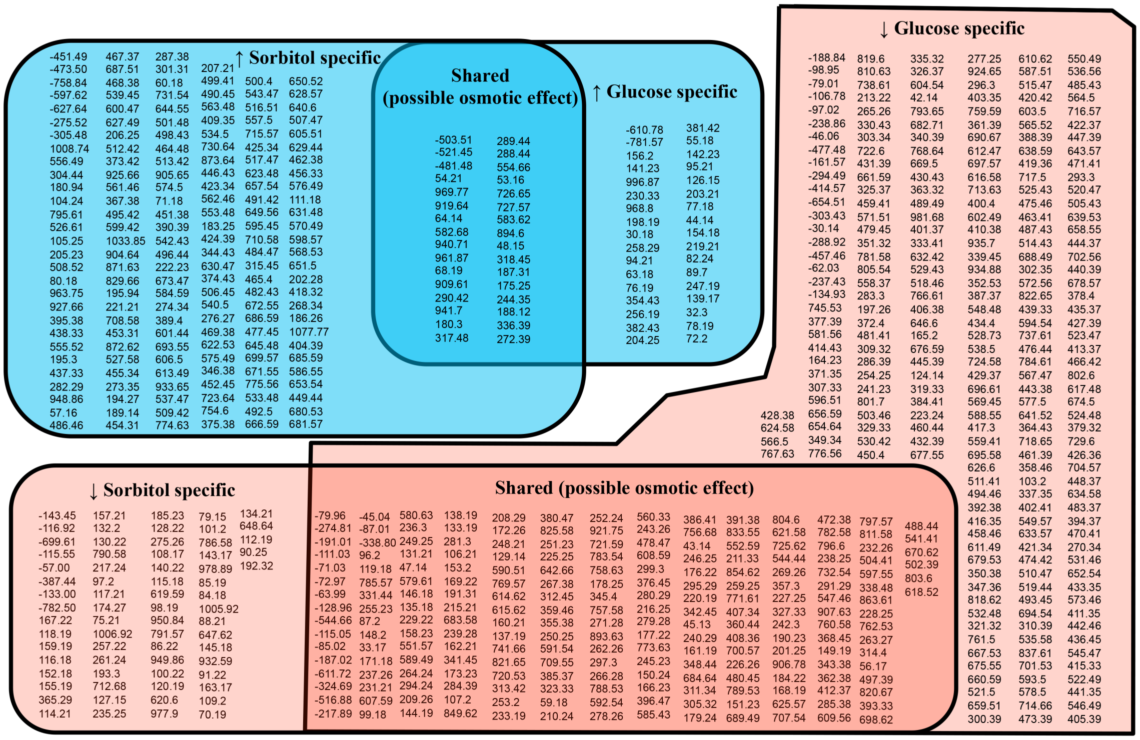
↑ Sorbitol specific
Shared
(possible osmotic effect)
↑ Glucose specific
↓ Glucose specific
↓ Sorbitol specific
Shared (possible osmotic effect)
