# Supplementary figures and images for: High levels of glucose alter Physcomitrella patens metabolism and trigger a differential proteomic response
Source: PLoS One. 2020 Dec 4;15(12):e0242919. doi: 10.1371/journal.pone.0242919 (PMC7717569; doi:10.1371/journal.pone.0242919)

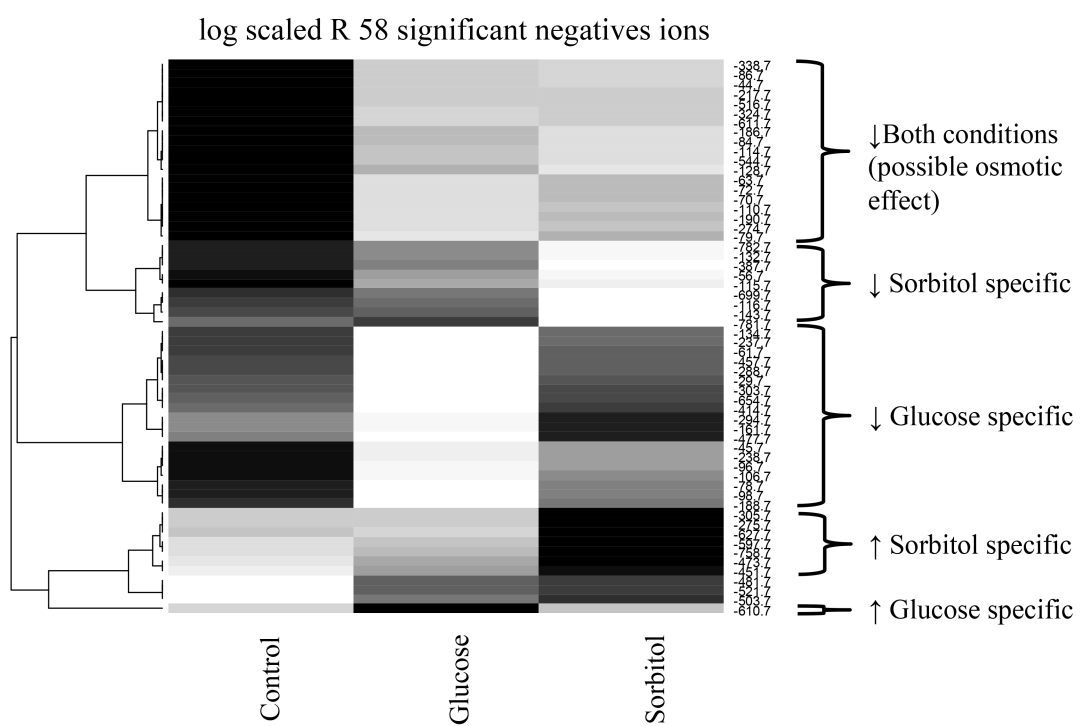

Supplement: S2 Fig — P. patens protonemata were exposed to 300 mM of either glucose or sorbitol for 24 h. An optimized hierarchical clustering based on correlation R was applied to 58 significant negative ions. The metabolomic fingerprint is represented as a grayscale barcode that depicted the relative intensity (ion abundance), black indicates high and white indicates low. Ion similarity is revealed by the left dendrogram. Results shown correspond to three independent biological samples. (PDF) [file pone.0242919.s002.pdf]

log scaled R 50 top intensity significant positives ions

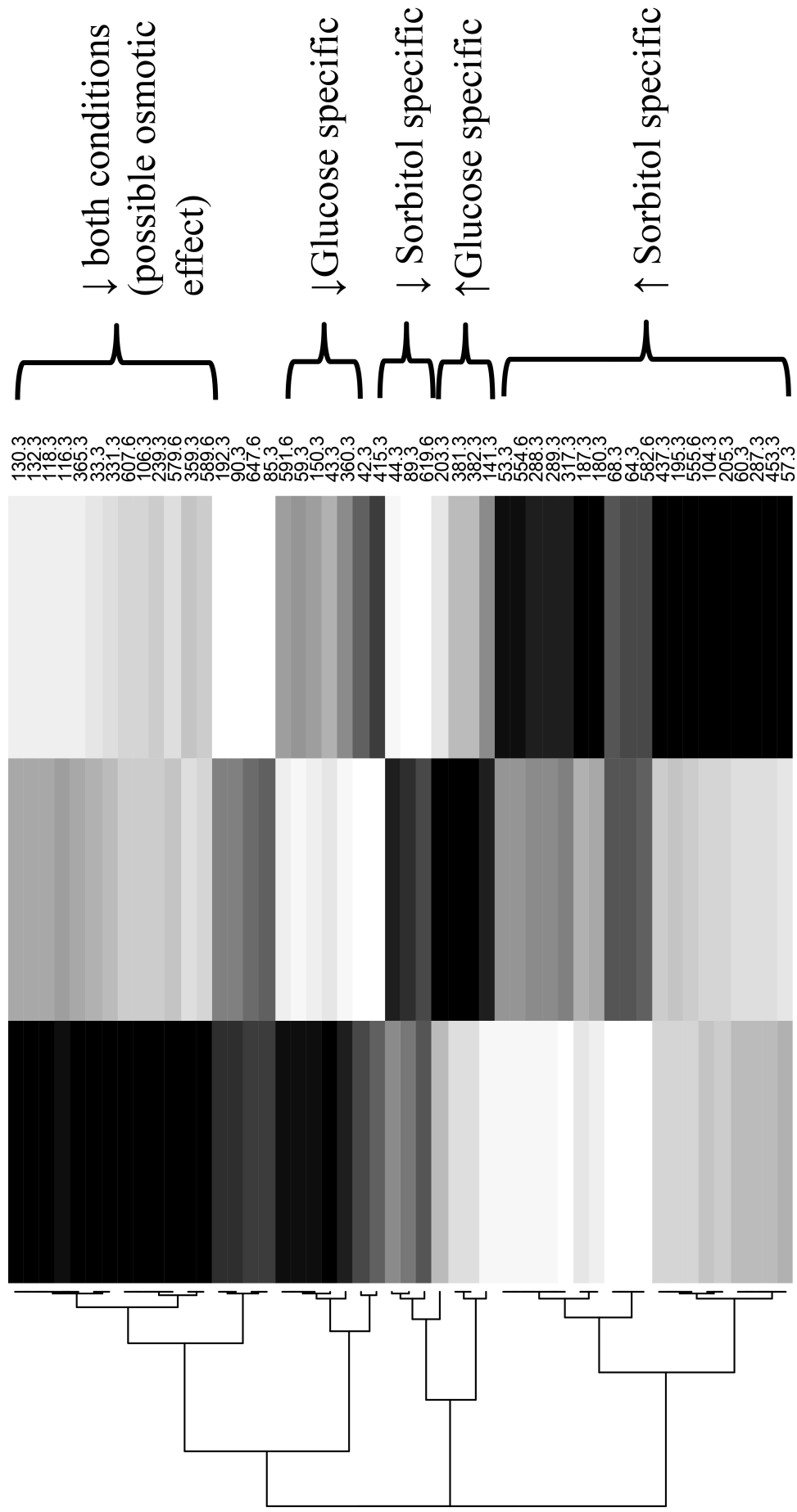

Supplement: S3 Fig — P. patens protonemata were exposed to 300 mM of either glucose or sorbitol for 24 h. An optimized hierarchical clustering based on correlation R was applied to 50 top intensity significant positive ions. The metabolomic fingerprint is represented as a grayscale barcode that depicted the relative intensity (ion abundance), black indicates high and white indicates low. Ion similarity is revealed by the left dendrogram. Results shown correspond to three independent biological samples. (PDF) [file pone.0242919.s003.pdf]

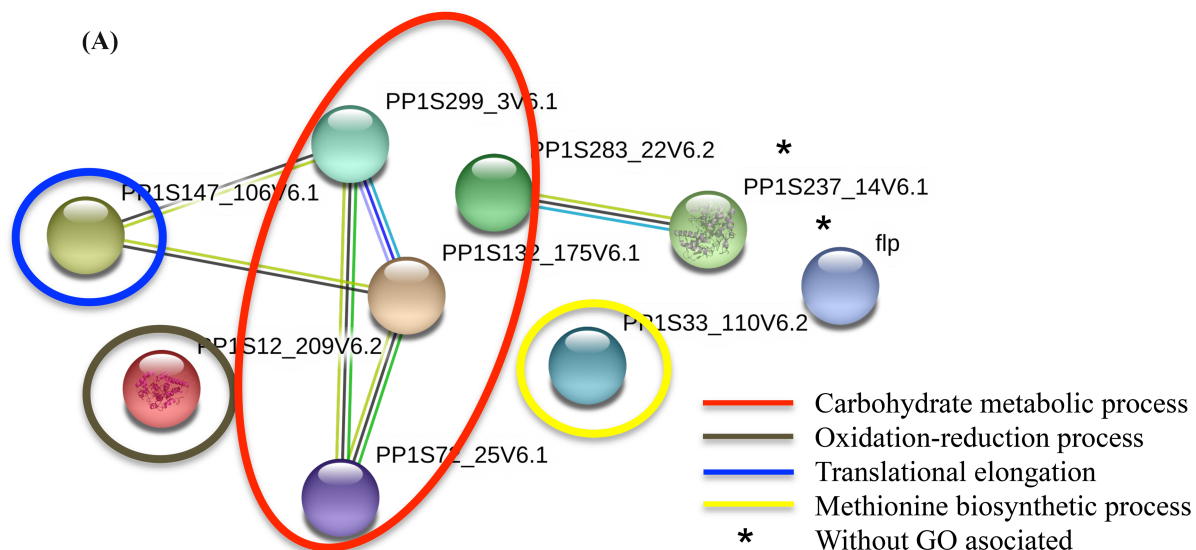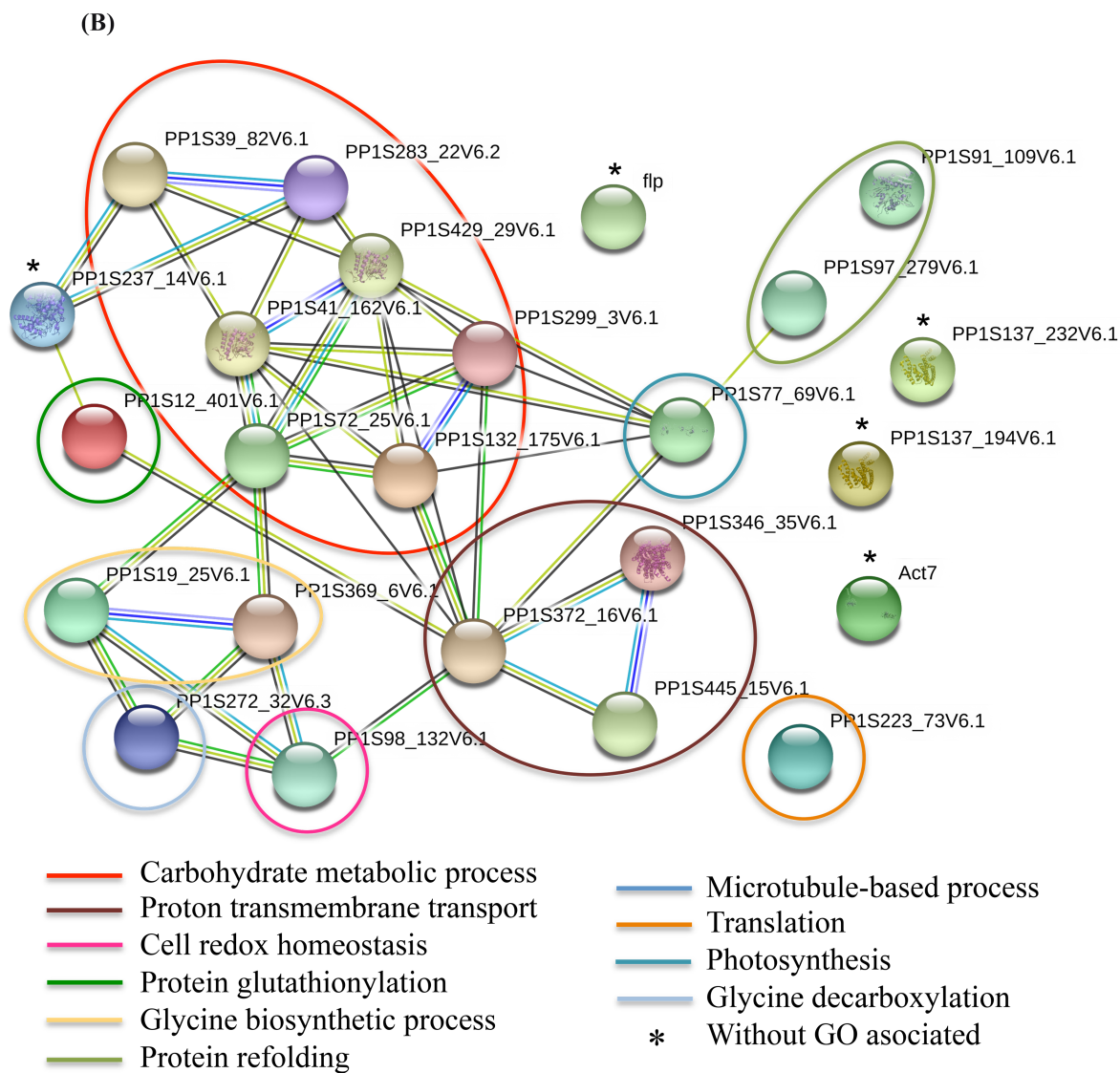

Supplement: S4 Fig — (A) Analysis of proteins relatively less abundant in response to glucose. (B) Proteins relatively less abundant in response to sorbitol (Q8H932 protein was not shown by the STRING database analysis). The lines connecting proteins are; Cyan, curated databases; magenta, experimentally determined; green, gene neighbourhood; red, gene fusions; blue, gene co-occurrence; light green, textmining; black, co-expression; mauve, protein homology. Colored circles highlight biological processes. (PDF) [file pone.0242919.s004.pdf]
